# Independently evolved extracellular electron transfer pathways in ecologically diverse *Desulfobacterota*

**DOI:** 10.1093/ismejo/wraf097

**Published:** 2025-05-17

**Authors:** Dario R Shaw, Krishna P Katuri, Veerraghavulu Sapireddy, Olga Douvropoulou, Jeffrey A Gralnick, Pascal E Saikaly

**Affiliations:** Biological and Environmental Science & Engineering (BESE) Division, King Abdullah University of Science and Technology (KAUST), Thuwal 23955-6900, Makkah Province, Saudi Arabia; Biological and Environmental Science & Engineering (BESE) Division, King Abdullah University of Science and Technology (KAUST), Thuwal 23955-6900, Makkah Province, Saudi Arabia; Biological and Environmental Science & Engineering (BESE) Division, King Abdullah University of Science and Technology (KAUST), Thuwal 23955-6900, Makkah Province, Saudi Arabia; Biological and Environmental Science & Engineering (BESE) Division, King Abdullah University of Science and Technology (KAUST), Thuwal 23955-6900, Makkah Province, Saudi Arabia; BioTechnology Institute and Department of Plant and Microbial Biology, University of Minnesota, Twin Cities, St. Paul, MN 55108, United States; Biological and Environmental Science & Engineering (BESE) Division, King Abdullah University of Science and Technology (KAUST), Thuwal 23955-6900, Makkah Province, Saudi Arabia; Environmental Science & Engineering Program, King Abdullah University of Science and Technology (KAUST), Thuwal 23955-6900, Makkah Province, Saudi Arabia

**Keywords:** extracellular electron transfer, electron transport, *Desulfobacterota*, *Desulfuromonas*, electroactive bacteria, multiheme cytochromes, iron-reducing bacteria

## Abstract

Extracellular electron transfer plays a role in the biogeochemical cycling of carbon, metals, sulfur, and nitrogen, and has wide-ranging biotechnological applications. The metal-reducing (Mtr), outer-membrane cytochrome (Omc), and porin-cytochrome (Pcc) pathways facilitate electron transfer to insoluble electron acceptors via *trans-*outer membrane cytochrome complexes. Although these pathways perform a similar function, they are phylogenetically unrelated, indicating independent evolutionary origins. Here, we report an extracellular electron transfer mechanism in which the high-current producing bacterium *Desulfuromonas acetexigens* differentially co-expresses, at transcript and protein levels, the porin-cytochrome, outer-membrane cytochrome, and metal-reducing pathways, along with high-molecular-weight cytochromes containing a large number of hemes (up to 86 heme-binding motifs), under extracellular electron transfer growth conditions (i.e. electrode under set potential or naturally occurring iron oxide minerals as the electron acceptor). Additionally, we identified over 40 *Desulfobacterota* species from diverse ecological environments that encode the outer-membrane cytochrome and metal-reducing pathways, with the majority also expressing the porin-cytochrome pathway. The newly identified metal-reducing proteins in *Desulfobacterota* form a major lineage, greatly expanding the known diversity of these proteins. To our knowledge, *mtrCAB* genes have not been reported in the *Desulfobacterota* phylum (formerly classified as *Deltaproteobacteria*), nor has any electroactive organism been shown to express these phylogenetically distant pathways simultaneously. These findings have ecological implications, challenging the belief that certain extracellular electron transfer pathways are exclusive to specific taxa, and suggesting that these pathways are more widespread than previously thought. Additionally, this reveals a previously unrecognized versatility in microbial electron transfer mechanisms that can be exploited in biotechnological applications.

## Introduction

Microbial respiration via extracellular electron transfer (EET) is a major, widespread biological process in anoxic environments that contributes significantly to the global biogeochemical cycling of carbon, metals, sulfur, and nitrogen [1–9]. The EET process also has potential applications in bioremediation, production of renewable energy, biofuels, bioelectronics, biocorrosion, and sustainable wastewater treatment [5, 10–16]. Despite the importance of EET in a diverse range of environmental processes and biotechnology, the ecology and mechanisms that microbes use to perform respiration of insoluble electron acceptors are not yet well understood [3, 6]. In fact, most of what is known about EET has been derived from studies conducted with pure cultures of bacteria affiliated with the genus *Geobacter* (belonging to *Desulfobacterota*, formerly *Deltaproteobacteria*) and *Shewanella* (belonging to Gammaproteobacteria), which have served as model organisms to understand EET [3]. A common feature among these EET-capable bacteria is the presence of several multi-heme *c*-type cytochromes responsible for the electron flux [3, 5]. *Shewanella* spp*.* uses the metal-reducing (Mtr) pathway to transfer electrons to insoluble extracellular electron acceptors (i.e. minerals containing Fe(III) or electrode in bioelectrochemical systems) [3, 17]. In the Mtr pathway, electrons derived from the oxidation of carbon sources flow through intracellular menaquinol pools in the cytoplasmic membrane to the *trans-*outer membrane cytochrome complex MtrCAB, which mediates the electron transfer from the periplasm to extracellular electron acceptors [3, 18]. Similar to the mechanism in *Shewanella*, *Geobacter* spp*.* use electron conduits such as *trans-*outer membrane cytochrome complexes (Omc) and Pcc proteins to transfer electrons to extracellular electron acceptors [3, 19, 20]. Although Mtr, Omc, and Pcc pathways use *trans-*outer membrane complexes, they are phylogenetically unrelated, suggesting that these systems have evolved independently to mediate electron transfer across the bacterial surface using the same design principle [3, 19]. To the best of our knowledge, no bacterium has been reported to encode or use all these pathways in combination.

Despite the functional similarities of these extracellular electron transfer mechanisms, there are significant differences in the phylogenetic distribution of these pathways. The Omc pathway has been reported in members of the Geobacteraceae family, such as *Geobacter* spp [3]. In contrast, recent phylogenetic and comparative genomic analyses have revealed that the Mtr pathway is widely distributed across diverse bacteria from a broad range of taxonomic classifications and environments [21]. The analyses suggest that the *mtrCAB* genes have been extensively spread through horizontal gene transfer (HGT) events and subsequent vertical transmission [21]. In that study no members of the *Desulfobacterota* phylum (formerly classified as *Deltaproteobacteria*) were detected to encode the Mtr pathway despite sharing similar habitats and ecological niches with potential HGT donors of the *mtrCAB* genes. Within *Desulfobacterota*, members of *Desulfuromonadia* such as *Geobacter* spp. and *Desulfuromonas* spp. are distinctive for their ability to carry out a wide range of metabolic processes and perform EET [3, 5, 14].

*Desulfuromonas* spp. are widely present and have been reported and/or isolated from various anoxic environments. These include mud, iron-rich soils, brackish habitats, aquifers, groundwater, Antarctic sediments, hydrocarbon-contaminated sediments, uranium-contaminated sediments, sulfur-rich environments, deep subsurface brine, methane gas wells, hydrothermal vents, sub-seafloor sediments, deep subsurface environments, and marine and freshwater sediments [22–40]. This widespread presence provides evidence of the remarkable ability of these organisms to thrive in diverse environments. Additionally, *Desulfuromonas* spp. are also often found along with *Geobacter* spp. as part of the community in natural and engineered environments [22, 37, 41–43]. Due to their presence in a wide array of natural environments, *Desulfuromonas* spp. activity has been reported to contribute to the global cycling of carbon, sulfur, minerals, and nutrients [24, 25, 27, 36, 37, 43–45]. The activity of *Desulfuromonas* has been reported even in extreme environments such as (sub-)Antarctic sediments, in experiments using RNA-SIP with ^13^C-labeled acetate [27]. The authors highlighted the importance and activity of iron and sulfate reducers in these sediment communities [27]. In another study on groundwater systems, the transcript abundance of *Desulfuromonas* was positively associated with Fe^+3^, As^+3^, and NH_4_^+^. The authors suggested that N_2_ fixation might be tightly interconnected with N, Fe, and As transformations in these environments [24]. In hydrothermal vents, *Desulfobacterota* such as *Desulfovibrio, Desulfobulbus, Desulfobacteria,* and *Desulfuromonas* displayed the greatest abundance of sulfate-reducing genes. The authors underscored the relevance of carbon and sulfur cycling in hydrothermal ecosystems and highlighted the importance of microorganisms such as *Desulfuromonas* in these environments [36]. *Desulfuromonas acetexigens* is of particular significance because, besides being present in natural environments, it has been found as a dominant organism in engineered systems such as domestic wastewater, anaerobic sludge, raw paper mill effluents, and bioelectrochemical reactors [41, 42, 46–48]. In addition, electrochemical characterization and pure culture studies in bioelectrochemical systems revealed that *D. acetexigens* is capable of EET and electrode respiration, producing high peak current densities of ~10 A m^−2^ with acetate as the electron donor [46]. Despite contributing to global biogeochemical cycles, being widespread in natural and engineered environments, and producing high current densities in bioelectrochemical systems, the ecology, metabolic potential, and pathways involved in EET in *D. acetexigens* have not been systematically explored to date.

In this study, we integrate bioelectrochemistry, genomics, stimulus-induced comparative transcriptomics, and differential proteomics to investigate the metabolism and EET pathways of the high-current producing *D. acetexigens,* aiming to gain insights into what confers its ecological adaptive advantage and wide niche adaptation. Additionally, phylogenetic analyses were carried out to establish the distribution and ecological context of the co-occurrence of the phylogenetically distant EET pathways Mtr, Omc, and Pcc across the tree of life. This is the first systematic examination of the molecular mechanisms of EET in a member of *Desulfuromonas* spp. Our findings provide a robust foundation for future genetic and proteomic studies of this group of organisms, which exhibit ecological versatility, broad niche adaptation, and unique metabolic traits with potential for biotechnological applications.

## Materials and methods

### Cultivation of *D. Acetexigens*

*D. acetexigens* strain 2873 (DSM 1397) was obtained from the German collection of Microorganisms and Cell Cultures (DSMZ, Germany) as lyophilized culture and activated in the culture medium suggested by DSMZ. After activation, *D. acetexigens* was cultured at 30°C in 50 ml airtight, rubber septa-sealed anaerobic bottles containing 45 ml of DSM 148 growth medium. Prior to inoculation in microbial electrolysis cells (MECs), the strain was subsequently sub-cultured three times (3 days of incubation time per batch) in fumarate-containing DSM 148 growth medium. The growth medium was prepared by adding the following constituents: KCl 0.1 g L^−1^, NH_4_Cl 0.2 g L^−1^, Na_2_HPO_4_ 0.6 g L^−1^, CH_3_COONa 0.49 g L^−1^, 10 ml of vitamin mix (DSM 141), and 10 ml of trace elements solutions (DSM 141). The synthetic medium was boiled and flushed with N_2_/CO_2_ (80:20) for 1 h to remove the dissolved oxygen. Then, the pH of the medium was adjusted to 7 by adding NaHCO_3_ and was transferred to serum vials for final autoclaving at 121°C for 20 min and 15 psi. CaCl_2_ and MgSO_4_ solutions were autoclaved separately to avoid salt precipitation and were added to the sterile medium to a final concentration of 0.1 g L^−1^ and 0.4 g L^−1^, respectively. Degassed filtered-sterilized sodium fumarate solution was added to the medium to a final concentration of 8 g L^−1^ as the terminal electron acceptor to initiate growth after inoculation. All transfers were performed under an anaerobic environment.

### MECs construction, operation, and electrochemical analyses

In order to study the electroactivity of *D. acetexigens*, bioelectrochemical analyses were conducted in MECs. Cultures of *D. acetexigens* grown in serum vials were centrifuged at 5500 g for 8 min, and the resultant pellet was washed separately with saline phosphate buffer. Cell density was measured with flow cytometry and normalized to ~3 × 10^8^ live cells ml^−1^. The resultant cell pellets were used as inoculum for the MECs. To determine the potential at which *D. acetexigens* exhibits high electrochemical activity and current production, experiments were conducted in single-chamber MECs inoculated with *D. acetexigens* and operated at different potentials using 10 mM sodium acetate (CH_3_COONa) as the electron donor. The single-chamber MECs were made of glass reactors with a 500 ml working volume containing multiple working electrodes. The working electrodes (anodes) were graphite rods 8 cm in length (7.5 cm inside the reactor) and 0.5 cm in diameter. Platinum mesh was used as the counter electrode (cathode) and Ag/AgCl as the reference electrode (Bioanalytical Systems, Inc.). The multiple working electrodes were operated at set potentials of −0.1, 0, 0.1, 0.2, and 0.3 V vs. Ag/AgCl. The amperometric current was monitored continuously using a VMP3 potentiostat (BioLogic Science Instruments, USA), with measurements taken every 60 s and analyzed using EC-lab V 10.02 software.

Subsequent bioelectrochemical analyses were performed in triplicate single-chamber MECs made of borosilicate glass reactors with 300 ml working volume. The reactors had appropriate ports for placing electrodes, a gas collection bag, and for liquid sampling. Carbon cloth (6 cm × 6 cm) was used as a working electrode (anode). Platinum gauze (5 cm × 5 cm) was used as a counter electrode (cathode) and Ag/AgCl as a reference electrode (3.5 M KCl, Bioanalytical Systems, Inc.). The reactors were assembled with the electrodes positioned vertically, arranged ~3 cm apart from each other. Epoxy glue was used to seal every opening in the reactor to avoid leakage.

Gas bags (0.1 L Cali −5 -Bond. Calibrate, Inc.) were connected to the MECs to collect any gas generated. The gas composition in the gas bags was analyzed using a multiple-gas detection gas chromatograph (SRI 8610C gas chromatograph, SRI Instruments). Argon (99.9% purity) at a constant flow of 1 ml/min was used as the carrier gas. A 250 μl gas sample was injected into the GC for each analysis. Concentrations of individual gases were measured with a thermal conductivity detector (TCD) using a 6′ × 1/8" S.S. molecular sieve 13X packed column. The temperature ramp protocol consisted of the following: the initial temperature of 45°C was maintained during a 1.7 min run time to detect N₂, H₂, and CH₄. The column temperature then ramped to 100°C, maintaining a run time of 5 min to detect CO₂. The slope of the temperature gradient was 55°C/min. At the end of the sample run (i.e. 7.7 min), the temperature dropped to 45°C for subsequent sample injections. The inorganic medium composition in the MECs was the same synthetic growth medium (See Cultivation of *D. acetexigens* section above), but free of fumarate and with 10 mM sodium acetate as the sole carbon and energy source. The MECs were operated at a set potential of −0.1 V vs. Ag/AgCl. The amperometric current was monitored continuously using a VMP3 potentiostat (BioLogic Science Instruments, USA), with measurements every 60 s and analyzed using EC-lab V 10.02 software. The medium in the MECs was gently mixed with a magnetic stirrer throughout the course of the experiments. The pH of the MECs was not controlled but was always between 7.2–7.5. The MEC reactors were operated in fed-batch mode at 30°C in a controlled-temperature room. All inoculations and batch changes were done in an anaerobic glove box (Coy Laboratory Products; Grass Lake Charter Township, MI, USA).

Cyclic voltammetry (CV) at a scan rate of 1 mV s^−1^ was performed to the mature anodic biofilms in the MECs at different time intervals and at a peak amperometric current to determine the redox and electron transfer behavior. The CV scans ranged from −0.7 to 0.1 V vs. Ag/AgCl and were carried out using a VMP3 potentiostat (BioLogic Science Instruments, USA). To determine the presence of extracellular secreted redox mediators, the CVs were performed to cell-free filtrates (filtered using a 0.2 μm pore diameter filter) collected from the MECs and placed in separate sterile electrochemical cells.

### Comparative transcriptomic analysis of *D. Acetexigens* in response to external electron acceptor

Comparative transcriptomic analysis was conducted to assess the genetic basis of *D. acetexigens* with respect to the type (electrode vs. fumarate) and availability of external electron acceptor, and to identify the potential metabolic pathways involved in the EET process. Six MECs were operated in parallel under applied potential (i.e. current flow conditions and electrode as electron acceptor) as described above (See MEC construction, operation, and electrochemical analyses section). After mature biofilm formation and stable reproducible current production were observed, half of the biofilm electrodes were collected with sterilized scissors in the anaerobic chamber for transcriptomics analysis. After sampling, the operation of one set of triplicate reactors was switched to OCV mode (i.e. no external electron acceptor) and another set of triplicate reactors was switched to fumarate as electron acceptor. Samples were taken for transcriptomics analysis at 2 h for reactors operated in OCV mode and at 24 h for reactors operated with fumarate as the electron acceptor. Samples were stored in RNAlater Stabilization Solution (Invitrogen) until further processing. Approximately 1 cm^2^ of biofilm electrode was placed into the Bead beating lysing matrix E tube of the PowerBiofilm RNA Isolation kit (QiAGEN). 100 μl of phenol:chloroform:isoamy alcohol pH 6.5–8.0 was added to the Bead beating Tube. Samples were physically disrupted with the following conditions: 2 × 40 s at 6 m s^−1^ in a FastPrep-24 (Mpbio) with 3 min resting time between cycles. After centrifugation at 13 000 g for 1 min at room temperature, the supernatant was transferred to a clean 2 ml collection tube and total RNA was extracted according to the manufacturer’s instructions. All samples were processed on the same day to reduce batch effects. The RNA concentration of all samples was measured in duplicate using the Qubit BR RNA assay. The RNA quality and integrity were confirmed for selected samples using TapeStation with RNA ScreenTape (Agilent Technologies). Potential residual DNA was removed using DNAase MAX kit (MoBio Laboratories Inc.) according to the manufacturer’s instructions. The samples were depleted of rRNA using the Ribo-zero Magnetic kit (Illumina Inc.) according to the manufacturer’s instructions. After rRNA depletion and DNase treatment, the samples were cleaned and concentrated using the RNeasy MinElute Cleanup kit (QIAGEN) and successful rRNA removal was confirmed using TapeStation HS RNA Screentapes (Agilent Technologies). The samples were prepared for sequencing using the TruSeq Stranded Total RNA kit (Illimina Inc.) according to the manufacturer’s instructions. Library concentrations were measured using the Qubit HS DNA assay and library size was estimated using TapeStation D1000 ScreenTapes (Agilent Technologies). The samples were pooled in equimolar concentrations and sequenced on a HiSeq2500 System (Illumina) using a 1 × 50 bp Rapid Run. Raw sequence reads in fastq format were trimmed using USEARCH [49] v10.0.2132, −fastq_filter with the settings -fastq_minlen 45 -fastq_truncqual 20. The trimmed transcriptome reads were also depleted of rRNA using BBDuk [50] with the SILVA database version 138.2 as the reference database [51]. The reads were mapped to the predicted protein coding genes of *D. acetexigens* DSM 1397 genome (VJVV00000000.1) generated from Prokka [52] v1.12 using USEARCH [49] v10.0.2132 with the following settings -usearch_global -id 0.98 -otutabout -strand both. Reads with a sequence identity below 0.98 were discarded from the analysis. The count tables were imported to R [53], processed and normalized using the DESeq2 workflow [54], and then visualized using ggplot2. Analyses of overall sample similarity were done using normalized counts (log transformed), through vegan [55] and DESeq2 [54] packages. Differentially expressed genes were evaluated for the presence of N-terminal signal sequences, transmembrane spanning helices (TMH) and subcellular localization using SignalP 5.0 [56], TMHMM 2.0 software, and PSORTb 3.0.2 [57], respectively. Differentially expressed genes that appeared annotated as “hypothetical” were reconsidered for a putative function employing BLAST searches (i.e. BLASTP, CD-search, SmartBLAST), MOTIF search, COG and PFAM databases, as well as by applying the HHpred homology detection and structure prediction program (MPI Bioinformatics Toolkit).

### Differential proteomics analysis of *D. acetexigens* in response to different insoluble external electron acceptors

Differential protein expression levels were evaluated by comparing *D. acetexigens* cultures incubated with iron oxide minerals (i.e. hematite, goethite, magnetite) and *D. acetexigens* biofilm on the electrode of MECs, against cultures grown with a soluble electron acceptor (i.e. fumarate). *D. acetexigens* cultures were incubated in triplicate in septa-sealed anaerobic vials containing 45 ml of growth medium with 10 mM sodium acetate as the electron donor and 15 mM of hematite, goethite, or magnetite as the sole electron acceptor. The serum vials and medium were prepared as described above (see *Cultivation of D. acetexigens* section). Soluble iron was determined using the ferrozine method as described elsewhere [58]. Incubations of *D. acetexigens* with the iron oxides as the electron acceptor were collected at 48 hours for protein extraction and proteome sequencing. Triplicate MECs with *D. acetexigens* biofilm on the electrode were operated at an applied potential of −0.1 V vs. Ag/AgCl. The MECs with *D. acetexigens* biofilm were constructed and operated as described above (see *Microbial Electrolysis Cells (MECs) construction, operation, and electrochemical analyses* section). Electrode biofilm samples were collected after mature biofilm formation and stable reproducible current production were observed. The biofilm electrodes were collected with sterilized scissors in the anaerobic chamber for proteomic analysis. *D. acetexigens* cultures grown with fumarate as the soluble electron acceptor were used as a control for the differential proteomic analyses. Protein extraction was performed as described in the *Proteomic analysis of D. acetexigens’* section of the Supplementary Materials and Methods. The proteome of the samples was characterized by nano-liquid chromatography–tandem mass spectrometry (LC–MS/MS) analysis. The peptide mixtures were analyzed using an Exploris 480 MS. Data analysis was conducted using Spectronaut software (version 18.5.231110.55695) in directDIA mode. The amino acid sequences of protein-coding genes from the *D. acetexigens* genome (GenBank accession number: VJVV00000000.1) were used as the reference database. Carbamidomethylation of cysteine was set as a fixed modification, while oxidation of methionine and acetylation of protein N-termini were set as variable modifications. The false discovery rate (FDR) thresholds were set to 1% for peptide-spectrum matches (PSMs), peptides, and proteins. Spectronaut software was used for data normalization and analysis. Protein abundances between samples were then compared using statistical tests to determine differential abundance.

### XPS analysis

X-ray photoelectron spectroscopy (XPS) analysis was performed to identify the depletion of Fe(III) on the surface of iron oxide minerals after the incubations. Samples were taken after 48 hours of incubation of *D. acetexigens* with the iron oxides as the electron acceptor and centrifuged at 7200 g for 20 minutes. The supernatant was discarded, and the pellet was dried in a vacuum oven to avoid any oxidation due to oxygen contamination. The samples were analyzed using the X-ray photoelectron spectrometer Kratos AXIS Ultra DLD (Kratos Analytical, UK) to obtain survey and high-resolution spectra. The data was further analyzed with Casa XPS software (Casa Software Ltd, UK) as described elsewhere [59].

### Sequence retrieval, alignment, and phylogenetic reconstruction of Mtr proteins

To search for additional Mtr proteins, we constructed a hidden Markov model (HMM) from alignments of Mtr sequences, including those of *D. acetexigens*, using hmmbuild in the HMMER v3.3.2 package with default parameters [60]. The HMM was scored against all bacterial genomes in the GTDB database as of March 20, 2025, using the hmmsearch function of the HMMER package with a score threshold of 100. Additionally, we performed conserved domains search by using HHpred [61] and DELTA-BLAST (Domain Enhanced Lookup Time Accelerated BLAST) [62] against all bacterial genomes in the NCBI database as of March 20, 2025. After retrieving the sequences, the proteins were manually curated. Genomes lacking the full set of MtrCAB proteins, duplicate proteins, proteins missing Mtr domains, proteins with an incorrect number of cytochromes, and those with additional protein domains as identified by NCBI's Conserved Domain Database search tool were excluded from further analyses. Amino-acid alignments were done with MAFFT [63] and PRANK algorithms [64]. Unreliable sequences, residues, and columns of the alignments were identified and eliminated with GUIDANCE2 using default parameters [65]. The alignments were visualized using Geneious Prime software version 2022.2.2, Jalview 2.11.1.4 [66] and WebLogo [67]. Individual maximum likelihood phylogenetic trees were constructed for each set of Mtr proteins using IQ-TREE v1.6.12 [68] with the following options: -m MFP -bb 1000 -alrt 1000. The empirically selected rate model for all Mtr sequences was based on Bayesian information criteria (BIC) as determined by ModelFinder [69]. Branch support was estimated using ultrafast bootstrapping [70] with 1000 bootstrap replicates. The final trees were visualized using iTOL [71]. Bacterial genomes that co-encode *mtr* and *omc* genes were subjected to phylogenomics analysis. HMM profiles for 71 single-copy core genes were concatenated using the Anvi’o platform [72]. The phylogenetic tree was estimated with IQ-TREE v1.6.12 as described above.

### TEM of *D. acetexigens*

*D. acetexigens* was grown under anaerobic conditions at 30°C in DSM 148 growth medium supplemented with acetate (10 mM) and fumarate (40 mM), as described above (See *Cultivation of D. acetexigens* section) [73]. Bacterial cell pellets were harvested after reaching the stationary phase, carefully washed with 0.1 M phosphate buffer (PBS), and fixed in a 2.5% glutaraldehyde solution containing PBS (50 mM, pH 7.4) for 24 h. The samples were then stained with 1% OsO4 and subjected to a series of alcohol dehydration steps (20%, 50%, 80%, and 100% ethanol). Next, the samples were embedded in low-viscosity epoxy resin and cured at 60°C for 24 h. Ultrathin sections (60–80 nm) were prepared using a Leica EM UC6 Ultramicrotome (Leica Microsystems GmbH, Germany) and placed on a carbon-coated copper grid. The grids were negatively stained with 2.5% uranyl acetate and visualized using a FEI Tecnai 12 TEM (FEI Company) operated at 120 keV.

### Structural prediction of Mtr and Omc proteins

De novo predicted structures for the Mtr and Omc proteins of *D. acetexigens* were generated using AlphaFold v2.3.2 [74] with default parameter settings. Heme motifs were visualized and inserted post-prediction at the conserved cysteine residues without modifying the original predicted folding. The predicted protein structures were searched against the crystal structures available in the PDB database to identify the best hit and were subsequently subjected to alignment and structural superposition using Foldseek with default parameters [75].

### Statistics and reproducibility

Three biological replicates were used for electrochemical, differential transcriptomic, and proteomic experiments. No statistical methods were used to predetermine the sample size. Statistical analyses for the comparative transcriptomics analyses were carried out in R [53] v. 3.3.4 using the R-studio environment. The comparative transcriptomics analysis is entirely reproducible using the R files available on https://github.com/DarioRShaw/EET_Desulfuromonas_acetexigens. Parameters and complete datasets generated from MASCOT, Proteome Discoverer, and Spectronaut analyses are available as supplementary information.The mass spectrometry proteomics data have been deposited to the ProteomeXchange Consortium via the PRIDE partner repository [76] with the dataset identifier PXD034195 and project DOI 10.6019/PXD034195.

## Results & discussion

### Electroactivity of *D. acetexigens*

**Figure 1:**
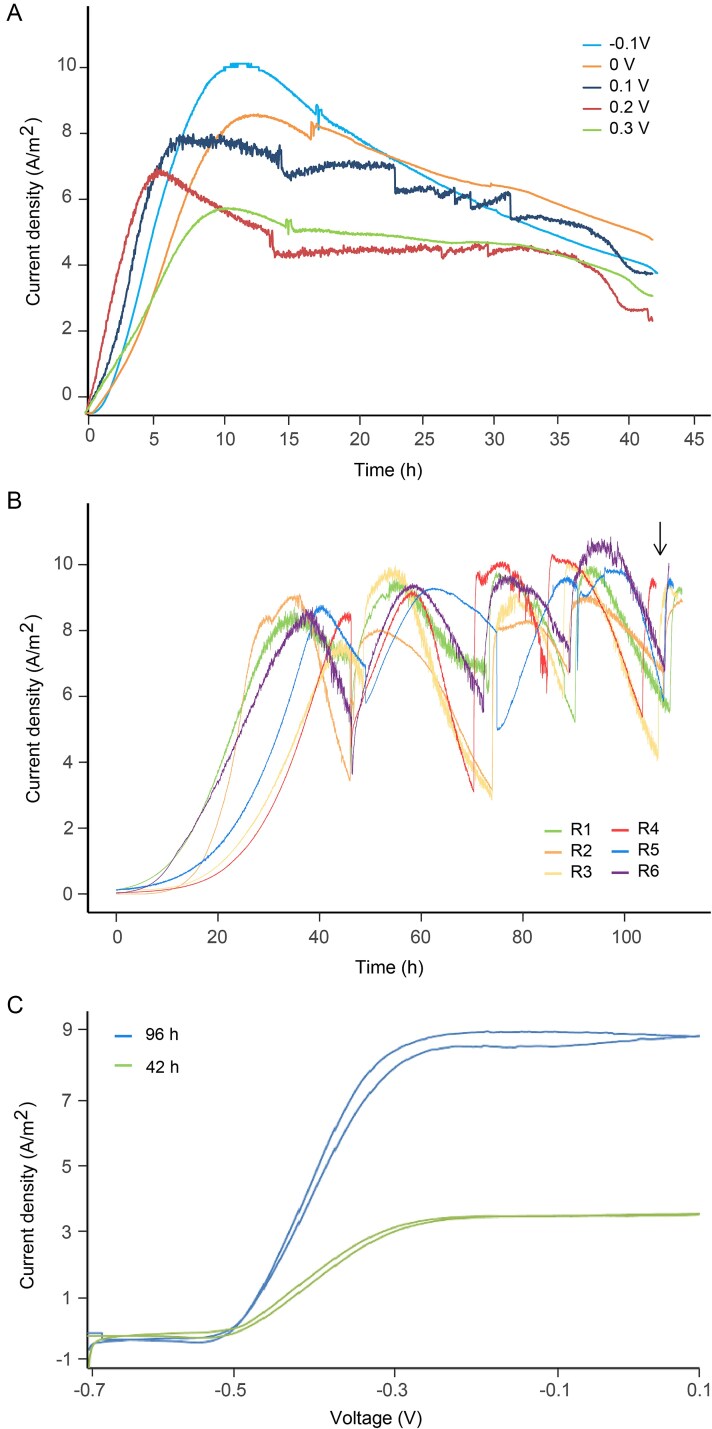
**Electroactivity of *D. Acetexigens***. (**A**) Chronoamperometry of a single-chamber multiple working electrode MEC inoculated with *D. Acetexigens* and operated under different set potentials. (**B**) Chronoamperometry of single-chamber MECs inoculated with *D. Acetexigens* and operated in fed-batch mode at a set potential of −0.1 V vs Ag/AgCl. The black arrow indicates the sampling point for differential expression analyses. (**C**) cyclic voltammetry (CV) response (1 mV s^−1^ scan rate) of *D. Acetexigens* at different time intervals of batch cycle operation with acetate as the electron donor and a set potential of −0.1 V vs Ag/AgCl.

Measuring the electrophysiological activity of bacteria is crucial to understand how they respond to different redox potentials and changes in the environment, specifically changes in electron acceptor. The EET activity of *D. acetexigens* was evaluated in single-chamber microbial electrolysis cells (MECs) operated at five different potentials (from −0.1 to 0.3 V vs Ag/AgCl) using acetate as the electron donor. The chronoamperometric response of *D. acetexigens* in the anode revealed rapid biofilm formation and current production within 10 hours after initial inoculation (Fig. 1A). The highest current production was observed at −0.1 V vs Ag/AgCl. Therefore, further electroactivity measurements were conducted at this potential. The chronoamperometric response of *D. acetexigens* cells was evaluated in six single-chamber MECs with electrodes poised at an applied potential of −0.1 V vs Ag/AgCl as the sole electron acceptor. The performance of the six MECs showed high reproducibility in current density with average peak currents of ~8 ± 1 A m^−2^ at the end of the first batch of operation (Fig. 1B). Cycles of rapid rise in current density after acetate feed, followed by a period of constant operation and a sharp fall due to acetate depletion, were reproducibly observed throughout the MEC operation (Fig. 1B). Further improvement in the maximum current density during the batch-feed cycles was observed, with peak current densities reaching a maximum of ~10 A m^−2^ over the ~100 h growth period in the MECs. These results are consistent with a previous study that evaluated the MEC performance in monocultures of *D. acetexigens* [41]. The current produced by *D. acetexigens* is comparable to that generated by the model organism *Geobacter sulfurreducens*. For instance, *G. sulfurreducens* KN400, a strain known for producing among the highest current densities in pure cultures, generates currents of around ~7.6 A m^−2^ [77]. The rapid start up, growth profile, and the magnitude of current generation are evidence of the EET potential of *D. acetexigens* biofilms.

Slow-scan cyclic voltammetry (CV) was used to correlate current density with biofilm age in developed *D. acetexigens* biofilms at different time intervals and with cell-free filtrates (filtered reactor solution). *In-situ* CV of *D. acetexigens* biofilm electrodes was performed at two time intervals (42 h and 96 h) following inoculation. The CVs exhibited a sigmoidal-shaped current-potential response, evidencing a direct EET mechanism and contact between bacterial cells and the electrode (Fig. 1C). Additionally, the current increase observed in the chronoamperometry (Fig. 1B) is mirrored by a current increase in the steady-state region in the CV response (Fig. 1C). Further examination of the first derivative of the CVs revealed the presence of a dominant redox transition with a half-wave potential of approximately −0.4 V vs. Ag/AgCl. These results are consistent with a previous study, which obtained a similar slow-scan CV response and estimated half-wave potential for *D. acetexigens* [46]. The *in situ* CV analyses provided evidence of the microbial-electrocatalytic oxidation of acetate by the *D. acetexigens* biofilm on the working electrode surface, supporting an electron transfer mechanism from the biofilm to the electrode through a dominant redox species. The possibility of extracellular redox mediator molecules and their involvement in current generation in *D. acetexigens* biofilms was also investigated. The CV analysis of the reactor’s spent wash solution was performed in separate MECs. The CVs with cell-free filtrates did not show changes in current density or display redox peaks (Supplementary Fig. 1), indicating that soluble redox mediators are not involved in the EET of *D. acetexigens* biofilms. Therefore, the observed electroactivity of *D. acetexigens* in the electrochemical analyses is due to a direct EET mechanism and contact between bacterial cells and the electrode.

### E‌ET potential of *D. acetexigens*

The study of *D. acetexigens* genome and proteome uncovered its metabolic versatility and a diverse collection of previously uncharacterized accessory genes, including *c*-type cytochromes, highlighting their potential for electron transfer (Supplementary discussion, Supplementary Fig. 2 & 3, Supplementary Tables 1–6). However, the presence of cytochromes alone is insufficient to determine their involvement in EET. Also, the large collection of cytochromes in *D. acetexigens* makes it challenging to identify those involved in EET. Therefore, to gain preliminary insights into the EET potential of *D. acetexigens* and to identify the cytochromes that might be involved in electron transport to insoluble extracellular electron acceptors (i.e. electrode), we conducted a comparative transcriptomics analysis. We used electrodes for our preliminary analyses of EET because electrodes are used solely for bacterial respiration, unlike metal oxides, which make it difficult to differentiate between metal oxide reduction for nutritional acquisition and respiration through EET activity. Differential expression was evaluated between *D. acetexigens* biofilms growing in MECs operated under an applied potential of −0.1 V vs. Ag/AgCl and after switching to a soluble electron acceptor (i.e. fumarate) or open circuit voltage (OCV) mode (absence of an electron acceptor). The potential of −0.1 V vs. Ag/AgCl was used because we observed the highest current production at this set potential (Fig. 1A). After quality control, filtering, and trimming of the RNA reads, between 67 and 81% of the reads mapped to the contigs of the *D. acetexigens* genome in all the samples (Supplementary Table 7). A transcriptome-wide analysis of gene expression revealed that biological replicate samples clustered together and were separated between experimental conditions as expected (Supplementary Fig. 4A). Additionally, the heatmap and hierarchical clustering of sample-to-sample distances showed high intra-replicate clustering and correlation, while inter-replicate correlation was low (Supplementary Fig. 4B). These results indicate high similarity between the biological replicates and suggest differentially expressed genes across the experimental setups (i.e. operation under set potential, operation with fumarate as the electron acceptor, and OCV operation mode). A transcriptome-wide analysis of the fold changes between the experimental conditions as a function of the average expression of each gene revealed differentially expressed genes when MEC operation was changed from set potential to fumarate (Supplementary Fig. 4C) or set potential to OCV mode (Supplementary Fig. 4d). In contrast, the overall gene expression between early and mature biofilm of *D. acetexigens* did not show differentially expressed genes (Supplementary Fig. 5), suggesting similar global gene expression at different growth stages when the electrode was used as the electron acceptor.

As expected, the majority of genes that showed differential expression in response to the change from electrode to fumarate as the electron acceptor were mainly associated with energy conservation and fumarate metabolism (Supplementary Table 8). Electrons derived from the oxidation of organics enter the inner-membrane quinone pool via the activity of primary dehydrogenases such as NADH dehydrogenase, succinate dehydrogenase, and electron transfer flavoproteins-associated with cytochrome *b* [78, 79]. In *G. sulfurreducens* PCA, inner membrane multiheme *c*-type cytochromes ImcH, CbcL, and CbcBA are required for respiration to extracellular electron acceptors at specific ranges of redox potentials [78, 79]. Our analyses revealed that *D. acetexigens* significantly expressed ImcH (TRO80643.1) and CbcL (TRO80642.1) homologs when the electrode was the electron acceptor (Supplementary Table 9). Electrons from the oxidation of menaquinol in the inner membrane are transferred to periplasmic *c*-type cytochromes. In known EET mechanisms such as those in *G. sulfurreducens* and *Shewanella oneidensis*, low molecular weight cytochromes act as periplasmic electron carriers [80]. Similarly, we identified low molecular weight electron carriers such as a PpcA homolog (TRO80283.1) in *D. acetexigens* (Supplementary Table 9). Outer membrane protein complexes can transfer the electrons from the periplasm to the bacterial surface via an electron transport chain [3]. Our analyses revealed the expression of OmcS homologs (TRO82312.1, TRO82315.1, TRO82316.1) in *D. acetexigens* (Supplementary Table 8), which are known to play a critical role in bacterial growth on insoluble electron acceptors in *G. sulfurreducens* [81]. Additionally, *D. acetexigens* significantly expressed *Geobacter* spp. homologs OmcZ (TRO81739.1), OmcO (TRO82434.1), and OmcE (TRO79781.1) when the electrode was the electron acceptor (Supplementary Table 9).

Our electrochemical analyses revealed that, despite most cells in the mature biofilm not being in contact with the anode, there was a direct correlation between biomass on the anode and current production (Fig. 1C). Additionally, soluble mediators were not found to play a role in current production (Supplementary Fig. 1), suggesting that redox mediators are not involved in the EET of *D. acetexigens* biofilms. These findings indicate the presence of a long-distance EET mechanism, enabling electrons to be transported through several bacterial layers in the *D. acetexigens* biofilm to the electrode. Recent studies have shown that *G. sulfurreducens* forms distinct appendages composed of OmcS, OmcE, or OmcZ, which facilitate long-distance EET [73, 81, 82]. Transmission electron microscopy (TEM) analyses revealed that *D. acetexigens* also produce appendages (Supplementary Fig. 6A), which can be distinguished from the characteristic single polar flagellum of *Desulfuromonas* species (Supplementary Fig. 6B) [38]. However, it remains uncertain whether these appendages are conductive or capable of facilitating long-distance EET. Comprehensive characterization of electrically conductive appendages is challenging due to the complex regulatory relationships between type IV pili and extracellular cytochrome production [73] and falls beyond the scope of this study. Nevertheless, de novo structural predictions of the expressed OmcE, OmcZ, and OmcS proteins of *D. acetexigens* were performed [74]. The predicted structural composition and length of the OmcE, OmcZ, and OmcS proteins in *D. acetexigens* were determined with a high level of confidence (i.e. per-residue measure of local confidence (pLDDT) estimates >90) (Supplementary Fig. 7a, c, f). Structural similarity alignment against resolved protein structures in database collections [75] revealed that the Cryo-EM resolved structures of OmcE, OmcZ, and OmcS nanowires from *G. sulfurreducens* were the best matches to the OmcE, OmcZ, and OmcS proteins of *D. acetexigens*, showing high levels of topological similarity (i.e. TM-score values >0.5, which corresponds to proteins with the same fold) (Supplementary Fig. 7b, d, g). Future studies should focus on characterizing the appendages produced by *D. acetexigens* to determine whether they are electrically conductive and to evaluate the roles of OmcE, OmcZ, and OmcS in long-distance EET mechanisms in this bacterium.

It is known that *G. sulfurreducens* encodes multiple Pcc electron conduits (i.e. OmcBC, ExtABCD, ExtEFG, or ExtHIJKL) implicated in electron transfer across the outer membrane [20]. These multiple electron conduits appear to have overlapping roles and are necessary for Fe(III) and Mn(IV) reduction [20]. Our analysis revealed that when the electrode was the sole electron acceptor, *D. acetexigens* significantly expressed multiple porin cytochromes clusters simultaneously. In addition to the expression of Omc proteins, a whole porin cytochrome cluster ExtHIKL (TRO80499.1-TRO80503.1) was also significantly expressed (Supplementary Table 9).

**Figure 2:**
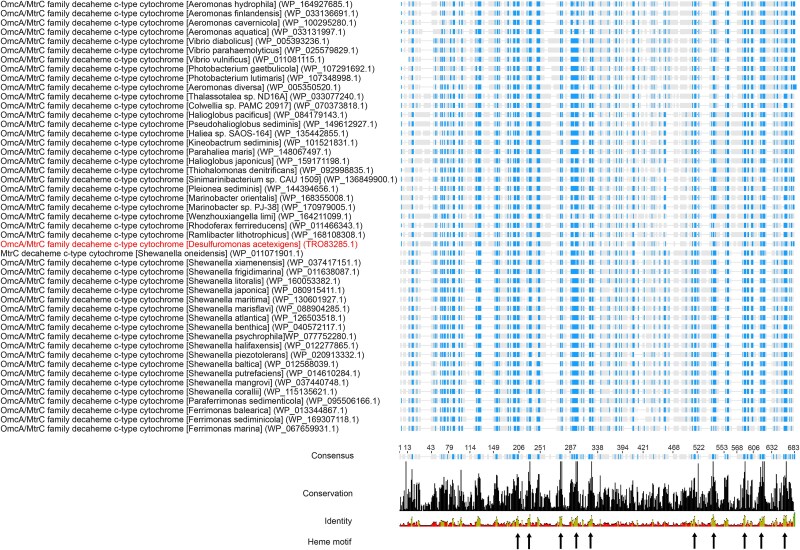
**Sequence alignment of outer membrane OmcA/MtrC family decaheme *c*-type cytochromes from various taxonomic groups**. Representative sequences from a multiple sequence alignment of MtrC family decaheme proteins are shown. The name of each species appears in brackets, followed by the accession number for each protein in parentheses. MtrC from *D. Acetexigens* appears highlighted. Residues with conservation >60% in the sequences appear in color. Arrows indicate the heme binding sites, which correspond to conservation levels >90%. The complete alignment of the Omc/MtrC family decaheme proteins is available in Supplementary Fig. 8.

**Figure 3:**
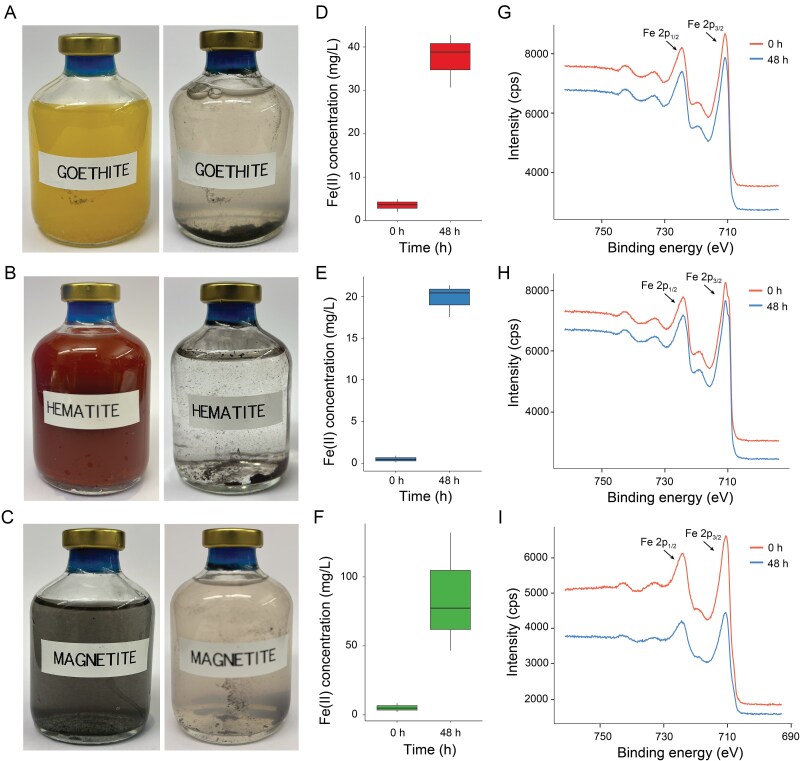
**Incubations of *D. acetexigens* with iron oxides as the sole electron acceptor for growth**. (**A–C**) Photographs of vials inoculated with *D. acetexigens* and using acetate as the electron donor at 0 h (left) and 48 h (right) after incubation with goethite (**A**), hematite (**B**), and magnetite (**C**). (**D–F**) iron (II) concentration in the medium at 0 h and 48 h after incubation of *D. acetexigens* with goethite (**D**), hematite (**E**), and magnetite (**F**). **G–I** XPS spectrum of the iron oxides before and after the incubation with goethite (**G**), hematite (**H**), and magnetite (**I**). The arrows indicate the Fe 2p_1/2_ and Fe 2p_3/2_ peaks characteristic of the iron oxides [90].

Our analysis revealed that *D. acetexigens,* which belongs to the *Desulfobacterota* phylum (formerly classified as *Deltaproteobacteria*), significantly expressed the metal reducing (Mtr) respiratory pathway, which is commonly found in phylogenetically distant *Shewanella* spp. (belonging to the Gammaproteobacteria class). The Mtr pathway in *Shewanella* spp. consist of a 10-heme cytochrome (MtrA) embedded in a 26 strand β-barrel (MtrB) in the outer-membrane lipidic environment. MtrAB forms a complex with an extracellular 10-heme cytochrome (MtrC), exposing its hemes over a large surface area for electrical contact with extracellular redox partners, such as metals and electrode [83]. When the electrode was used as the sole electron acceptor, *D. acetexigens* expressed a complete Mtr pathway (Supplementary Table 9). A homolog of the inner membrane tetraheme cytochrome CymA (TRO83644.1) was significantly expressed when the electrode was used as the electron acceptor. In *Shewanella* spp., CymA and its redox partners Fcc and CctA, mediate the transfer of electrons from the menaquinol to the periplasmic environment [84]. Similar to CymA, *D. acetexigens* significantly expressed homologs of periplasmic cytochrome MtrA (TRO83921.1), outer membrane β-barrel protein MtrB (TRO83920.1), and outer membrane MtrC family decaheme *c*-type cytochrome (TRO83285.1) when the electrode was used as the electron acceptor (Supplementary Table 9). In *Shewanella* spp., MtrCAB cytochromes offer overlapping redox potentials, facilitating a thermodynamic downhill electron transport chain to metals and electrode [85]. It is intriguing to see *D. acetexigens* encoding and expressing the Mtr pathway genes at the transcriptomic level (Supplementary Table 9), despite being phylogenetically distant from *Shewanella spp*. In *D. acetexigens*, the *mtrAB* genes were clustered together. However, *mtrC* is found in a different operon along with cytochrome biogenesis proteins and a transposase, challenging the paradigm that all the *mtr* genes are vertically or horizontally transferred in single clusters. Therefore, future studies should focus on mutant and gene knockout of *D. acetexigens* Mtr genes to investigate if the Mtr pathway in *D. acetexigens* plays a similar role in EET-based respiration as in *Shewanella* spp. Recently, a study found evidence that genes encoding the Mtr EET pathway have been disseminated largely through horizontal gene transfer (HGT), followed by vertical transmission, and are present in a broad diversity of bacteria found in a wide range of environments [21]. Our analysis revealed that the gene for the extracellular decaheme MtrC of *D. acetexigens* (TRO83285.1) is encoded right next to a transposase (TRO83286.1) in the same operon, supporting the hypothesis of HGT. Similarly, a transposase (TRO79784.1) is encoded downstream in an operon encoding a copy of MtrA (TRO79809.1) and *c*-type cytochrome maturation proteins (TRO79786.1, TRO79787.1). To evaluate the level of conservation of the Mtr protein sequences found in *D. acetexigens*, alignments were constructed for each of the Mtr proteins alongside reference sequences that cover all reported and known diversity of MtrCAB to date. Protein alignment of the OmcA/MtrC family decaheme *c*-type cytochromes highlighted an overall conservation >60% between *D. acetexigens* and the reference MtrC sequences (Fig. 2). The positions and amino acid identities of the catalytic heme binding sites were the most conserved, with identity levels >90%. MtrA and MtrB alignments also showed high level (>60%) of conservation between *D. acetexigens* and the reference sequences (Data not shown). However, conservation estimates alone do not ensure that the protein will fold into a functional conformation. Nonetheless, to predict the structural conservation and similarity of MtrA, MtrB, and MtrC proteins of *D. acetexigens*, a de novo structural prediction was performed. A snapshot of the Mtr protein structures of *D. acetexigens* is shown in Supplementary Fig. 9. The resulting structural composition and length of the predicted Mtr proteins of *D. acetexigens* share a similar overall architecture to those obtained from crystal resolved structures of MtrCAB (Supplementary Fig. 9a, c, f) [83]. Sensitive protein structure alignment against resolved protein structures in database collections [75] identified the Mtr complex of *Shewanella baltica* (Protein Data Bank (PDB) 6R2Q) as the best-matching structure to the MtrA, MtrB, and MtrC proteins of *D. acetexigens*. Moreover, structural superposition of the MtrCAB model and 6R2Q structures showed alignment of the heme binding sites (Supplementary Fig. 9b, d, g). Although our data strongly support structural similarity between the newly identified Mtr proteins of *D. acetexigens* and the reported resolved structures of Mtr proteins, the exact substrate specificity of these proteins cannot be conclusively determined from these analyses alone. Future work will be required to confirm the exact substrate specificities and activity of these proteins. However, considering the metabolic and ecological context in which these proteins were expressed in our experiments (i.e. when the electrode was the electron acceptor), we hypothesize their functionality in EET.

### E‌ET pathways in *D. acetexigens* under different insoluble external electron acceptors

Comparative transcriptomics analysis revealed that *D. acetexigens* simultaneously expresses different pathways for EET, including a phylogenetically distant Mtr pathway. However, it is uncertain if these pathways are expressed at the protein level under EET conditions. Therefore, we performed a differential proteomic analysis to evaluate if the proposed EET pathways are also expressed at the protein level. Additionally, we aimed to assess the relevance of the newly identified Mtr pathway in the context of EET and determine if it is expressed under various natural and engineered insoluble external electron acceptors. Differential protein expression levels were evaluated by comparing *D. acetexigens* cultures incubated with iron oxide minerals (i.e. hematite, goethite, or magnetite) and *D. acetexigens* biofilms growing in MECs at an applied potential of −0.1 V vs Ag/AgCl, against cultures grown with a soluble electron acceptor (i.e. fumarate). Hematite, goethite, and magnetite were used for the experiments as they are the most abundant and widespread iron oxides in natural environments, and offer a continuum and variety of redox potentials [86–89]. The incubations of *D. acetexigens* with mineral oxides as the sole electron acceptor exhibited precipitation of the minerals, suggesting reduction after 48 hours of incubation (Fig. 3A, B, C). This reduction was confirmed by an increase in soluble iron (Fe II) in the medium (Fig. 3D, E, F) and XPS analysis showing depletion of Fe (III) at the mineral surface (Fig. 3G, H, I), indicating the use of the minerals by *D. acetexigens* as the electron acceptor. Abiotic incubations did not show mineral precipitation or iron reduction (Supplementary Fig. 10).

**Figure 4:**
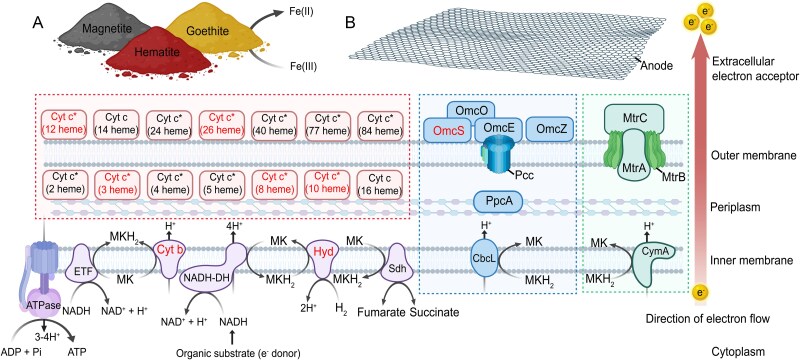
**Molecular model of EET in *D. acetexigens***. The EET metabolic pathways utilized by *D. acetexigens* to deliver electrons to external insoluble electron acceptors were constructed with proteins that showed a significant response (*P* < 0.05) to the iron oxides and electrode as the electron acceptor. The green-dashed box (box on the right) indicates the homolog metal-reducing (Mtr) pathway with *Shewanella* spp. The blue-dashed box (box in the center) indicates the homolog EET pathway with *Geobacter* spp. The cytochromes that are exclusively found in *Desulfuromonas* spp. are shown within the red-dashed box (box on the left). The black arrows indicate the direction of the reactions and proton (H^+^) transport across membranes. The red arrow indicates the direction of the electron flow to the external electron acceptor. Protein names in red indicate proteins found in multiple copies. Protein names marked with an asterisk (*) have undefined intracellular location, which could be either periplasmic or in the outer membrane. Identifiers of the proteins used in the model are listed in Supplementary Table 16.

The differential proteomics analysis revealed the simultaneous significant (*P* < 0.05) expression of multiple pathways for EET (Omc, Pcc, and Mtr), along with unique cytochromes of *D. acetexigens* at the protein level under all the insoluble electron acceptors tested (Supplementary Tables 12–15). Proteins associated with energy metabolism, cytochromes, and EET were among the most differentially expressed proteins across all tested conditions, representing the top 10% of all significantly expressed proteins (Supplementary Tables 12–15). In particular, the Omc and Mtr pathways were consistently within the top 5% of significantly expressed proteins under EET conditions. Overall, the protein expression profiles obtained confirm the results from the differential transcriptomics analyses. The conditions in which the expression was tested (insoluble electron acceptor as the sole electron acceptor for growth) support the hypothesis that the newly identified Mtr pathway is involved in EET, demonstrating the capability of *D. acetexigens* to synthesize and employ this pathway that is phylogenetically distant to Omc and Pcc pathways. Based on the differential proteomic analyses (Supplementary Tables 12–15), a model was developed to describe the EET pathway utilized by *D. acetexigens* to deliver electrons to insoluble electron acceptors (Fig. 4). The model includes proteins significantly upregulated (*P* < 0.05) under all the EET conditions tested. The list of proteins used in constructing the EET pathway and their identifiers can be found in Supplementary Table 16. Considering the absence of a genetic system for knockout experiments in *Desulfuromonas* spp., the proposed EET model provides a basis for genetic studies and offers functional insights for future research.

### Phylogenetic relationship among MtrCAB sequences across bacteria

**Figure 5:**
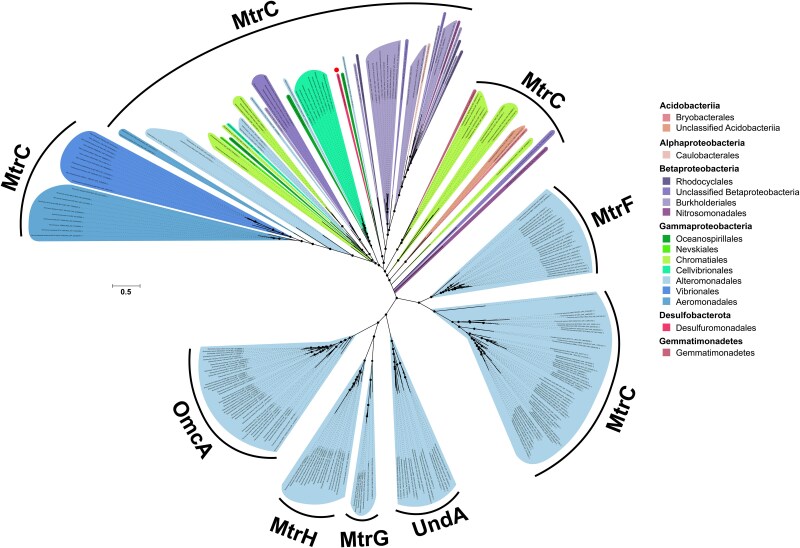
**Phylogenetic relationship among known OmcA/MtrC decaheme *c*-type cytochromes from diverse bacteria and the newly identified *D. acetexigens* MtrC detected in this study**. Maximum-likelihood phylogenetic tree constructed from a concatenated alignment of the known diversity of OmcA/MtrC decaheme *c*-type cytochromes and the *D. acetexigens* MtrC detected in this study. Color codes were assigned by taxonomic order. Bootstrap support values ≥90% from 1000 resamples are indicated with filled black circles. The scale bar represents average changes per amino acid position. MtrC, OmcA, MtrH, MtrG, UndA, and MtrF indicate members of the MtrC family of proteins. MtrC of *D. acetexigens* is indicated with a red dot. A full resolution image of the tree is available in Supplementary Fig. 11.

A phylogenetic analysis was conducted to understand the taxonomic relationship between the newly identified Mtr proteins expressed by *D. acetexigens* and the known diversity of Mtr proteins to date. Individual phylogenies were constructed for MtrC, MtrA, and MtrB protein sequences. The Phylogenetic reconstructions of the Mtr proteins largely agree in overall topology, and all the reconstructions show that *D. acetexigens* Mtr proteins and the reference sequences are closely related phylogenetically (Fig. 5, Supplementary Fig. 12 & 13). The MtrC protein sequence of *D. acetexigens* was found to be phylogenetically distant from other members of the MtrC family of proteins (i.e. OmcA, UndA, MtrG, MtrH) and the paralog MtrF. Additionally, MtrA and MtrB protein sequences were found to be phylogenetically distant from the Mtr gene cluster paralogs (i.e. MtrD, MtrE). These results suggest a closer evolutionary relationship of the *Desulfuromonas* Mtr proteins to MtrCAB rather than to other members of the MtrC protein family or gene paralogs of the Mtr cluster. The results of the MtrCAB maximum likelihood trees showed different clades with some phylogenetically diverse clusters (Fig. 5, Supplementary Fig. 12 & 13). Similar to previous phylogenetic reconstructions of Mtr sequences [21], the relationships between MtrCAB proteins are incongruent with the species phylogeny. The overall topology, diversity, and widespread occurrence of MtrCAB across diverse, taxonomically distant bacteria suggest a combination of horizontal and subsequent vertical transmission, rather than vertical transmission alone as the sole agent driving Mtr distribution [21].

### Genetic co-occurrence of Mtr, Omc, and Pcc pathways in *Desulfobacterota*

**Figure 6:**
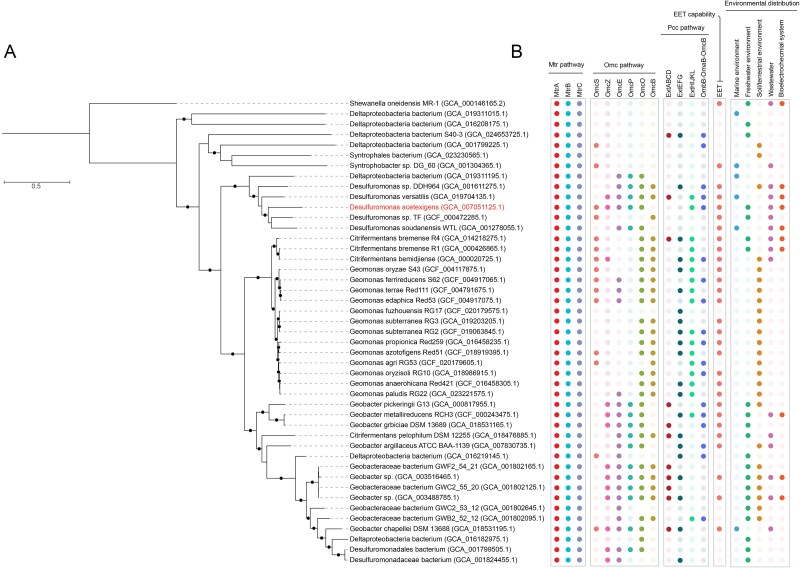
**Phylogenomic relationship and environmental distribution of bacteria that encode phylogenetically distant Mtr, Omc and Pcc pathways.** (**A**) Maximum likelihood phylogenetic tree constructed from 71 single-copy core genes of the *Desulfobacterota* genomes identified in this study encoding Mtr genes. Bootstrap support values ≥90% from 1000 resamples are indicated with filled black circles. The accession number for each genome is provided in parentheses. The tree was rooted using *Shewanella oneidensis* MR-1 genome as the outgroup. (**B**) Genetic occurrence of Mtr, Omc, and Pcc genes in the genomes identified in this study and their environmental distribution. Filled colored dots indicate the presence of a gene or set of genes in a genome or the presence of the organism in a given environment. “EET” indicates that the EET capability has been observed using graphene oxide (GO), Fe(III) oxides or electrode as the electron acceptor. Unfilled dots in “EET” indicate that the bacteria have not been tested for EET capability. “Wastewater” in environmental distribution indicates that the species has been observed in wastewater or wastewater treatment systems. “Bioelectrochemical system” in environmental distribution indicates the detection of the organism in electrode biofilms from bioelectrochemical reactors. Detailed characteristics and full description of the genomes, genes, and environments are found in Supplementary Table 17.

A previous study [21] revealed that the *mtrCAB* cluster is widespread across a broad range of taxonomic classifications but was not detected in members of the *Desulfobacterota* phylum (also known as *Thermodesulfobacteriota* and formerly classified as *Deltaproteobacteria* class [91]). As a result, the discovery of a conserved Mtr pathway in *D. acetexigens* was particularly intriguing. In some members of *Desulfobacterota* (i.e. *Geobacteraceae*, *Desulfuromonadaceae*), the phylogenetically distant Omc and Pcc pathways evolved independently to provide a similar function (i.e. EET) [3]. Our analyses also revealed the presence of the Omc and Pcc pathways in *D. acetexigens*. Based on these observations, we investigated whether the genetic co-occurrence of the Mtr, Omc, and Pcc pathways in a single organism extends beyond *D. acetexigens* to other organisms. We systematically screened all publicly available genomes in the NCBI database (as of March 20, 2025) across the entire tree of life for the presence of these pathways by targeting conserved domains of Mtr, Omc, and Pcc proteins. Although individual pathways were detected in diverse bacterial lineages, the concurrent presence of Mtr, Omc, and Pcc genes was uniquely restricted to members of the *Desulfobacterota* phylum. Additionally, we examined the environmental and ecological contexts of these organisms to better understand the niches associated with this metabolic configuration. In total, we identified 45 *Desulfobacterota* species besides *D. acetexigens* encoding both the Mtr and Omc pathways, as well as 34 species encoding one or more of the Pcc gene clusters (Fig. 6, Supplementary Table 17). The genes encoding the Mtr pathway are clustered together in the order of *mtrC*, *mtrA*, and *mtrB* in the *S. oneidensis* MR-1 genome [21]. In *Desulfobacterota* genomes analyzed, the *mtrAB* genes were consistently clustered together (Supplementary Table 17). However, the position of *mtrC* gene varied within the same cluster or genome. In some instances, *mtrC* was found in a different operon together with cytochrome biogenesis proteins and a transposase. The fact that *Desulfobacterota mtrC* was found in a different operon than *mtrAB* may have been one of the reasons why these proteins were not detected in previous analyses targeting the entire *mtrCAB* cluster [21]. Most of the identified bacteria encoding *mtr*, *omc,* and Pcc complex genes belong to the *Desulfuromonadia* class and were found in a wide range of environments, including freshwater and marine sediments, estuaries, subsurface brine, subsurface soils, minerals, hydrothermal vents, and engineered systems (Fig. 6, Supplementary Table 17). Of the identified species, 29 have been reported to perform EET to graphene oxide, metal oxides, or electrodes, or involved in direct interspecies electron transfer (Fig. 6, Supplementary Table 17). It is not known whether the identified microorganisms use the Mtr system for EET, or if the *Desulfobacterota* MtrC are associated with the MtrAB proteins. Additionally, the exact substrate specificities and activity of the *Desulfobacterota* Mtr proteins cannot be established from these analyses alone. However, *D. acetexigens* expressed the Mtr system at both mRNA and protein level under ecological conditions relevant to EET. Moreover, considering that the majority of bacteria identified encoding Mtr, Omc, and Pcc pathways in parallel are reported to perform EET to metal oxides or electrodes, we hypothesize that these newly identified genes may play a role in EET.

The underlying reasons that explain the presence of *mtr* genes in *Desulfobacterota* as well as why those genes are maintained in the genomes are not clear from the analyses in the current study. Previous studies suggest that the transfer of *mtr* genes depend on the donor’s phylogenetic compatibility along with the recipient’s ecology, physiology, and cell architecture [21]. For instance, all the known species that employ the Mtr pathway are exclusively Gram-negative bacteria [21], suggesting that the Mtr system is limited to bacteria possessing an outer membrane. The *Desulfobacterota* identified in this study encoding the *mtr* genes are present in environments that overlap with the habitats of known potential *mtr* donors and carriers, raising the possibility of horizontal gene transfer events and subsequent vertical transfer. For instance, *Geobacter* spp., *Shewanella* spp., and *Desulfuromonas* spp. have been reported to coexist in high abundance in marine and freshwater sediments [92–94]. It is important to consider that the *mtr* genes in the *Desulfobacterota* species identified in this study might be under a strong positive selection pressure, contributing to their preservation in the genomes of these species. It is known that metal oxide minerals exist in nature as a complex continuum of potential energies [88]. As diverse taxa participate in EET to minerals, competition between different organisms along any part of the energy continuum can be expected. Therefore, we hypothesize that having multiple EET pathways (i.e. Omc, Mtr, and Pcc) may provide a competitive advantage to these *Desulfobacterota* species by allowing the exploitation of a wider range of redox potentials in minerals and metal oxides [88].

**Figure 7:**
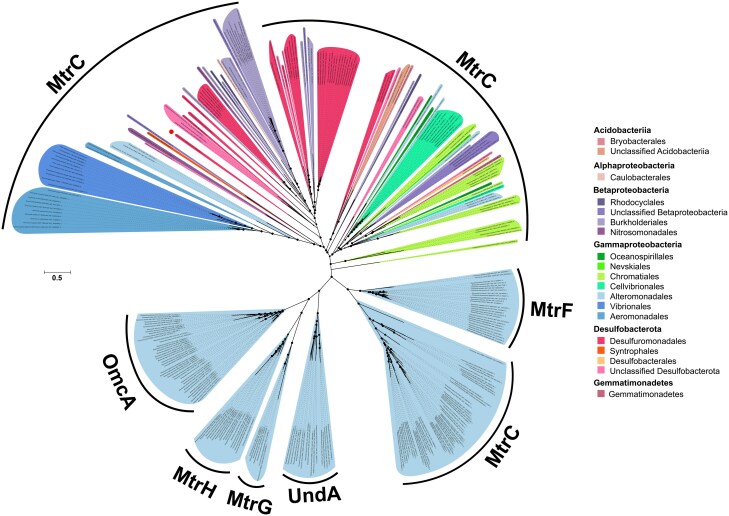
**Phylogenetic relationship among known OmcA/MtrC decaheme *c*-type cytochromes from diverse bacteria and the *Desulfobacterota* MtrC detected in this study**. Maximum-likelihood phylogenetic tree constructed from a concatenated alignment of the known diversity of OmcA/MtrC decaheme *c*-type cytochromes and the *Desulfobacterota* MtrC detected in this study. Color codes were assigned by taxonomic order. Bootstrap support values ≥90% from 1000 resamples are indicated with filled black dots. The scale bar represents the average changes per amino acid position. MtrC, OmcA, MtrH, MtrG, UndA, and MtrF indicate members of the MtrC family of proteins. MtrC of *D. acetexigens* is indicated with a red dot. Full resolution image of the tree is available in Supplementary Fig. 14.

Our analyses led us to discover that *mtr* genes are also present in several members of the *Desulfobacterota* phylum, indicating that they are more widespread than previously considered. To infer the evolutionary relationship of the newly identified *Desulfobacterota* Mtr sequences, individual phylogenies were constructed for MtrC, MtrA, and MtrB protein sequences. The different Mtr reconstructions largely agree in overall topology and support our newly identified sequences as a divergent major lineage (Fig. 7, Supplementary Fig. 15 & 16). Our results suggest that the Mtr pathway is more widespread than previously considered and significantly expands the known diversity of these proteins. This new lineage of Mtr proteins provides the foundation for further studies of these enzymes, and serves as a guide to prioritize genetic and protein characterization studies.

### Ecological implications

Taken together, this study provides a comprehensive insight into the extensive metabolic versatility of a widespread bacterium in natural and engineered environments, as well as the first in-depth examination of the EET molecular mechanism in a species of the *Desulfuromonas* genus, which are important players in the biogeochemical carbon, sulfur, and iron cycles [24, 25, 27, 36, 37, 43–45]. The unprecedented diversity of *c*-type cytochromes found in *D. acetexigens* highlights the vast potential for electron transfer in this organism. Among the expressed cytochromes, to the best of our knowledge, our study reports the expression of the largest cytochrome with the highest number of heme-binding motifs not in a complex, containing 86 heme-binding motifs, more than any previously reported cytochromes in electroactive bacteria. This discovery opens new avenues for understanding the electron transfer and storage metabolic capabilities conferred by these proteins. In addition, we show an unprecedent case of an organism that expresses in parallel, at both transcript and protein levels, the phylogenetically distant Mtr, Omc, and Pcc pathways, which are some of the most studied and prevalent EET pathways in electroactive organisms in nature and engineered systems. Given the metabolic and ecological context in which these proteins were differentially expressed in our experiments (i.e. when iron oxides and electrode were the electron acceptors), the evidence suggests that they may be involved in EET-based respiration. The multiple cytochromes and electron transfer pathways in *D. acetexigens* might play a role in the high current densities observed in the MEC reactors. A distinctive system that includes multiple phylogenetically distant electron conduits has not been previously documented in any electroactive organism and challenges the existing understanding of the EET mechanisms. However, this is not unexpected, considering that bacteria would evolve complex mechanisms to harness energy from diverse redox potentials found in minerals [88]. A previous study suggested that even under the most common laboratory conditions, electroactive bacteria such as *G. sulfurreducens* utilize multiple electron transfer pathways in parallel [88]. Also, synthetic biology efforts have attempted to enhance EET rates in *S. oneidensis* by incorporating pathways from other organisms [18, 95].

The discovery of a conserved Mtr pathway in *Desulfuromonas* was unexpected, as the presence of *mtrCAB* genes had not been previously reported or detected in the *Desulfobacterota* phylum. Multiple lines of evidence presented in the study support that these proteins are members of the Mtr family of proteins including: (I) differential expression at transcript and protein levels under EET conditions; (II) significant global homology, high-quality alignment, and phylogenetic relationship against all the known diversity of Mtr sequences reported to date; (III) high level of conservation of key residues required for electron transfer activity; and (IV) predicted structures of the newly identified Mtr proteins with similar fold and shared structural superposition of key functional residues with the Mtr crystal structures of *Shewanella* spp. Furthermore, our analyses identified phylogenetically distant Mtr, Omc, and Pcc pathways co-existing in over 40 species within the *Desulfobacterota* phylum from diverse ecological environments. These previously uncharacterized Mtr proteins form a major lineage group, evidencing that the Mtr system is more widespread and has greater prevalence across the tree of life. The identification of this lineage greatly expands the known diversity of Mtr proteins and has implications in the evolution and distribution of these enzymes within bacteria. The identified *Desulfobacterota* encoding the Mtr, Omc, and Pcc pathways in parallel occur in a variety of environments (e.g. soil, marine and freshwater sediments, deep ocean ecosystems). Additionally, the majority of these species have been reported to perform EET. Among the pathways and proteins identified in these species, we discovered a previously uncharacterized EET Mtr pathway in *Geobacter metallireducens*, one of the most extensively studied organisms in the field. These findings highlight that even within relatively well-studied microbial taxa, there are still unrecognized metabolic pathways and overlooked bacterial groups contributing to critical carbon and/or metal biogeochemical cycling functions.

The findings of this study also provide a solid foundation for future genetic, biochemical, and protein characterization analyses. Our findings offer a blueprint to prioritize the bacterial groups and proteins that might be involved in EET and may have potential applications in biotechnology, including bioremediation, energy recovery from waste streams, production of nanomaterials, bioenergy, and bioelectronic materials [3, 96, 97].

## Conclusions

The study of *D. acetexigens* led to the discovery of a novel EET mechanism involving the differential co-expression at transcript and protein levels of phylogenetically distant EET pathways (i.e. Pcc, Omc, and Mtr) under EET growth conditions, such as electrode at a poised potential or iron oxide minerals as electron acceptors. In addition to these EET pathways, we identified the differential expression of previously uncharacterized high-molecular-weight cytochromes with a high number of heme-binding motifs. Among these cytochromes, one cytochrome was found to contain 86 heme-binding motifs, which, to our knowledge, represents the highest number reported for any individual cytochrome in electroactive bacteria. Taken together, these findings provide the first systematic examination of the molecular mechanisms of EET in a member of the *Desulfuromonas* genus. The discovery of a conserved Mtr pathway in *Desulfuromonas* was unexpected, as the presence of *mtrCAB* genes had not been previously reported or detected in the *Desulfobacterota* phylum (formerly classified as *Deltaproteobacteria*). Therefore, phylogenetic analyses were conducted to determine how the three distinct EET pathways Mtr, Omc, and Pcc, are distributed across the tree of life. Our analyses revealed novel, divergent major lineages of Mtr proteins in *Desulfobacterota* from diverse environments and ecological contexts, along with the presence of Omc and Pcc pathways. The identification of Mtr proteins in the *Desulfobacterota* phylum suggests a greater prevalence of the Mtr mechanism and considerably expands the known phylogenetic diversity of these proteins. The co-occurrence of phylogenetically distant pathways within a single organism is unprecedented in the known biology of EET-capable microorganisms and highlights that even within relatively well-studied microbial taxa, there are unrecognized electron transfer metabolic pathways.

## Supplementary Material

Shaw_et_al_Supplementary_material_wraf097

Shaw_et_al_Supplementary_tables_1-17_wraf097

## Data Availability

Raw sequencing reads of genomic and transcriptomic data associated with this project can be found at the NCBI under BioProjects PRJNA552430 and PRJNA726843. Annotated GenBank files of *D. acetexigens* genome used in this study can be found under the accession number VJVV00000000. R files used for the comparative transcriptomics analysis are available on https://github.com/DarioRShaw/EET_Desulfuromonas_acetexigens. Complete Datasets generated in the differential expression analyses are available as supplementary information. The mass spectrometry proteomics data have been deposited to the ProteomeXchange Consortium via the PRIDE partner repository [76] with the dataset identifier PXD034195 and project DOI 10.6019/PXD034195. Parameters and complete datasets generated from MASCOT, Proteome Discoverer, and Spectronaut analyses are available as supplementary information.
