# Supplementary material for: Independently evolved extracellular electron transfer pathways in ecologically diverse Desulfobacterota
Source: ISME J. 2025 May 17;19(1):wraf097. doi: 10.1093/ismejo/wraf097 (PMC12133095; doi:10.1093/ismejo/wraf097)
Supplement: Shaw_et_al_Supplementary_material_wraf097 [file shaw_et_al_supplementary_material_wraf097.pdf]

Supplementary Materials

Independently evolved extracellular electron transfer pathways in ecologically diverse *Desulfobacterota*

Dario R. Shaw[1]*, Krishna P. Katuri[1]*, Veerraghavulu Sapireddy[1], Olga Douvropoulou[1], Jeffrey A. Gralnick[2], Pascal E. Saikaly[1,3]*

Affiliations:

[1]Biological and Environmental Science & Engineering (BESE) Division, King Abdullah University of Science and Technology (KAUST), Thuwal 23955-6900, Saudi Arabia

[2]BioTechnology Institute and Department of Plant and Microbial Biology, University of Minnesota, Twin Cities, St. Paul, Minnesota 55108, United States.

[3]Environmental Science & Engineering Program, King Abdullah University of Science and Technology (KAUST), Thuwal 23955-6900, Saudi Arabia

*Corresponding authors:

Dario R. Shaw, dario.rangelshaw@kaust.edu.sa

Pascal E. Saikaly, pascal.saikaly@kaust.edu.sa

Krishna P. Katuri, krishna.katuri@kaust.edu.sa

Supplementary Materials & Methods

Comparative genomics analysis and metabolic reconstruction of *D. acetexigens*

To disclose the metabolic potential of *D. acetexigens*, genome-wide analysis was performed by annotating each gene of *D. acetexigens* DSM 1397 genome (VJVV00000000.1) using BlastKOALA [1]. The metabolic pathways were reconstructed using the Clusters of Orthologous Genes (COG), Pfam, and Kyoto Encyclopedia of Genes and Genomes (KEGG) reference databases. A metabolic pathway was considered to be present in *D. acetexigens* if the complete set of enzymes necessary to perform a reaction or pathway was present. The electron transfer potential of *D. acetexigens* was examined by annotating the *c*-type cytochromes in the genome. Proteins were considered *c*-type cytochromes if their sequence contained at least one CXXCH motif for covalent heme binding, where X can be any amino acid except one of C, F, H, P or W [2]. Proteins with more than two occurrences of the CXXCH motif were considered as multiheme *c*-type cytochromes. Proteins were excluded as cytochromes if they had a predicted function that indicated that they were not involved in electron transport. Pangenomic analysis was performed to compare *D. acetexigens* DSM 1397 genome (VJVV00000000.1) against the model electroactive bacterium *Shewanella oneidensis* MR-1 (GCA_000146165.2) and *G. sulfurreducens* PCA (NC_002939). The pangenomic analysis was performed in Anvi'o platform [3], following the pangenomics/metapangenomics workflow [4].

Proteomic analysis of *D. acetexigens*

In order to gain preliminary insights into the set of proteins expressed by *D. acetexigens*, we conducted a quantitative proteomic analysis. The preparation of proteins and proteome sequencing were performed as described elsewhere [5]. *D. acetexigens* cultures grown in serum vials with fumarate as the electron acceptor were centrifuged at 13,000 g for 20 min and the resultant pellet was washed with

saline phosphate buffer. Pelleted cells were resuspended in lysis buffer containing 3-[(3-cholamidopropyl) dimethylammonio] propanesulfonate (CHAPS) (4%, w/v), Triton X-100 (2%, w/v), urea (7 M), thiourea (2 M), and dithiothreitol (60 mM). Resuspended cells were lysed for 1 h at 4°C in a bath sonicator with settings at 50% power, 15 seconds ON, 90 seconds OFF pulse intervals. After sonication, the solution was centrifuged at 25,000 g for 1 h at 4 °C, and the supernatant was recovered as soluble protein fraction. Protein concentrations from the resulting supernatants were determined using the Thermo Scientific Pierce BCA Protein Assay Kit. Samples were loaded onto SDS containing polyacrylamide gels (NuPAGE 4–12% Bis–Tris gel) and electrophoresed for 2 cm. Gel lanes were then cut into 5 gel slices, and de-stained with acetonitrile. Gel slices were subjected to reduction with 10 mM DTT and alkylation with 50 mM iodoacetamide and in-gel tryptic digestion overnight using trypsin in a 1:50 trypsin:protein ratio. The resulting trypsin-digested peptides were extracted in 0.1% formic acid (FA) and desalted using Sep-Pak C18 Vac cartridges. The samples were then subjected to nano-liquid chromatography–tandem mass spectrometry (nano-LC-MS/MS) analysis. The peptide mixtures were analyzed using an Orbitrap Fusion mass spectrometer (MS) (Lumos, Thermo Fisher Scientific) coupled with an UltiMate 3000 UHPLC (Thermo Scientific). The peptides were then separated by an EasySpray C18 column with a 95-min gradient at constant 300 nL min^{-1}, at 40 °C. A full MS scan was acquired with a mass range of 350–1500 m/z, and the precursor ions that were above a $2e^4$ threshold and carried multiple positive charges (2–6) were selected for MS/MS analysis with dynamic exclusion enabled. Peptide mass fingerprints were analyzed using MASCOT v2.8.0.1 and Proteome Discoverer software version 2.4 (ThermoFisher Scientific) using the amino acids sequences of protein-coding genes from *D. acetexigens* genome (GenBank accession number VJVV00000000) as the reference database. The following settings were used for peptide and protein identification: carbamidomethyl (Cys) as fixed modification, oxidation (Met), and deamidation (NQ) as variable modifications, minimal peptide length 6 amino acids and a maximum allowed false discovery rate of

1% at both the peptide and protein level. Proteins with at least one unique peptide were selected for further analysis. Protein abundances were calculated using the exponentially modified protein abundance index (emPAI) [6]. The proteome was subjected to further analysis by searching the detected proteins against the Clusters of Orthologous Genes (COG) and the Kyoto Encyclopedia of Genes and Genomes (KEGG) databases.

Supplementary discussion

Genetic potential of *D. acetexigens*

Previous studies have suggested that *D. acetexigens* shares physiological traits and a similar ecological niche with the model electroactive organism *G. sulfurreducens* [7, 8]. Both bacteria can colonize electrodes, form biofilms, and use electrodes as an electron acceptor. They both metabolize acetate as electron donor, exhibit similar bioelectrochemical behavior, and can generate high current densities via direct electron transfer mechanisms [7–9]. Additionally, preliminary analysis of the *D. acetexigens* genome, revealed that both bacteria share a variety of multiheme *c*-type cytochromes [10]. However, despite the physiological similarities and the availability of the genome sequence, little is known about the genetic and metabolic potential of *D. acetexigens*. To gain preliminary insights into the overall metabolism of *D. acetexigens*, a comparative pangenomic analysis was performed to estimate how much the *D. acetexigens* genome (VJVV00000000.1) differs from those of EET-capable organisms such as *G. sulfurreducens* PCA (NC_002939). The phylogenetically distant *S. oneidensis* MR-1 (GCA_000146165.2) was used as an outgroup for the pangenomic analysis. *G. sulfurreducens* and *S. oneidensis* were chosen for the analysis because they are model EET organisms and the most intensively studied electroactive bacteria to date. They play important roles in biogeochemical cycles,

possess the ability to colonize and interact with electrodes, produce high current densities in bioelectrochemical systems, have complete genome sequences available, and have genome-scale metabolic models available [11–13]. Pangenomic analysis revealed a total of 9,485 gene clusters, resulting in 13,406 genes calls among the three electroactive bacteria (Supplementary Fig. 2). Of the total 9,485 gene clusters, 2,077 have unknown function and lack clusters of orthologous groups of proteins (COGs) annotation. The gene clusters were categorized into six groups based on their occurrences across the three genomes (Supplementary Fig. 2): (1) unique gene clusters of *D. acetexigens*, (2) unique gene clusters of *G. sulfurreducens*, (3) unique gene clusters in *S. oneidensis,* (4) shared gene clusters between *D. acetexigens* and *S. oneidensis*, (5) shared gene clusters between *D. acetexigens* and *G. sulfurreducens*, and (6) core gene clusters (i.e., gene clusters present in all evaluated genomes). Interestingly, *D. acetexigens* had at least 2,206 unique gene clusters (i.e., gene clusters that are not present in the other analyzed genomes), which is comparable to the vast unique genetic potential of *G. sulfurreducens* (2,229 unique gene clusters) and *S. oneidensis* (3,936 unique gene clusters) (Supplementary Fig. 2b). As the shared gene content between genomes is an effective predictor of their phylogenetic and ecological relationships [4, 14, 15], we used the distribution of gene clusters to determine the relationships among the evaluated genomes. As expected, the analysis showed a closer evolutionary and ecological relationship between *D. acetexigens* and *G. sulfurreducens* than with *S. oneidensis* (Supplementary Fig. 2a). This relationship is evidenced by *D. acetexigens* sharing at least 1,779 gene calls with *G. sulfurreducens* and only 156 with *S. oneidensis* (Supplementary Fig. 2b). Even though the phylogenetic and ecological relationship between *D. acetexigens* and *G. sulfurreducens* is closer, the functional homogeneity index of the shared gene clusters between these bacteria is low (Supplementary Fig. 2a). This suggests that while the gap/residue distribution within a gene cluster is conserved, the identity and level of conservation of amino acid residues across the protein sequence are low. Only 192 gene clusters were shared between all three genomes (i.e., core gene clusters),

corresponding to single-copy gene clusters (SGC) (Supplementary Fig. 2a). All the single-copy core genes had known COG function and matched the set of genes present in most of known bacterial species, often used to estimate completeness and contamination in bacterial genomes (e.g., ribosomal proteins) [16]. The full list of bacterial SCGs has been described previously by Campbell *et al*., 2013 [17]. Our analysis, consistent with previous research on the *S. oneidensis* genome [18], revealed that the *S. oneidensis* genome is larger, has a higher number of gene clusters, more unique accessory genes, and less GC-content compared to *D. acetexigens* and *G. sulfurreducens* (Supplementary Fig. 2a). These results were anticipated since previous pangenomic analysis demonstrated that *Shewanella* strains possess extensive genome variability, high levels of gene gain, gene loss, horizontal gene transfer, and a high number of accessory genes [19]. Overall, the comparative genomic analysis showed that the vast genetic and functional diversity of *D. acetexigens* is comparable to those of the model EET organisms, *S. oneidensis* and *G. sulfurreducens*, and that *D. acetexigens* possess a large quantity of novel and unknown gene clusters that have not been studied before (Supplementary Fig. 2).

Cytochrome diversity of *D. acetexigens*

Multiheme *c*-type cytochromes are key for electron transport and the respiration of insoluble extracellular electron acceptors in Gram-negative bacteria [20]. In addition, cytochrome diversity within a bacterial population can provide insights into the organism's ecological role and electron transfer capability . Similar to characterized members of the family *Geobacteraceae*, *D. acetexigens* appears to excel in its ability to exchange electrons with the extracellular environment [7, 21]. However, despite the genome announcement of *D. acetexigens* [10], there has been no in-depth analysis of the available cytochromes in this bacterium. Therefore, to assess the diversity of cytochromes, the *D. acetexigens* genome was scanned for the heme-binding motif (CXXCH) of *c*-type

cytochromes. In addition, we determined the proteome of *D. acetexigens* to further verify the presence of the cytochromes at the protein level. Proteins were considered *c*-type cytochromes if their sequence contained at least one heme-binding motif. The genome announcement of *D. acetexigens* reported the presence of 39 putative *c*-type cytochromes [10]. However, our analysis revealed the expression of at least 89 *c*-type cytochromes (excluding cytochromes *b,* precursors, and cytochrome *c* biogenesis proteins) in *D. acetexigens* (Supplementary Tables 1-3). More than half of the cytochromes expressed by *D. acetexigens* are multiheme, lack annotation, do not have obvious homologs, or have not been characterized before, which might explain why they were undetected in previous annotation attempts [10]. The genetic potential for electron transport in *D. acetexigens* is comparable to that of *G. metallireducens* (90 genes encoding for *c*-type cytochromes) and *G. sulfurreducens* (104 genes encoding for *c*-type cytochromes) [22]. Additionally, *D. acetexigens* exhibits a greater number of *c*-type cytochromes in its genome compared to other reported *Desulfuromonas* species [23, 34], indicating a higher potential for electron transport. A previous study reported homologs of *Geobacter* spp. periplasmic cytochrome PpcA, inner membrane cytochrome ImcH, and outer membrane *c*-type cytochromes OmcZ, OmcI, and OmcO in *D. acetexigens* [23]. Our analysis also revealed the presence of these proteins in the genome and their expression at the protein level (TRO80283.1, TRO80643.1, TRO81739.1, TRO83921.1, TRO82434.1, respectively). Additionally, we identified multiple copies of the homolog of the outer membrane *c*-type cytochrome OmcS (TRO82312.1, TRO82315.1, TRO82316.1), which is required for Fe(III) and electrode respiration in *Geobacter* [25, 26] (Supplementary tables 1-3). *D. acetexigens* expressed a series of complex *c*-type cytochromes, highlighting its metabolic complexity. We detected the expression of cytochromes with predicted heme-binding motifs as high as 22 (TRO78690.1), 26 (TRO82309.1, TRO78417.1), 35 (TRO82434.1), 77 (TRO83203.1), and even up to 86 hemes (TRO81949.1) (Supplementary tables 1-3). The high number of heme-binding motifs of these proteins surpasses the previously reported complex

cytochromes of *Desulfuromonas* (up to 69 hemes) [24] and *Geobacter* species (34-37 hemes) [22]. The unprecedented quantity and diversity of *c*-type cytochromes found in *D. acetexigens* may reflect the importance of electron transport to this organism and suggest flexibility in the electron transfer mechanisms [27].

Metabolic pathways in *D. acetexigens*

To gain insights into functional diversity and reveal the specific pathways present in *D. acetexigens*, a metabolic reconstruction was performed by annotating each gene of the *D. acetexigens* genome using KO identifiers/KEGG orthologs as references. Additionally, a high-throughput proteomic analysis was conducted to verify the expression of these metabolic pathways and identify the set of proteins expressed by *D. acetexigens*. A metabolic pathway was considered present in *D. acetexigens* only if the complete set of enzymes necessary for the reaction or pathway was present. A graphical overview of the metabolic reconstruction and the full list of enzymes and metabolic pathways present in *D. acetexigens*, with their genomic identifiers, can be found in Supplementary Fig. 3 and Supplementary Tables 4-6, respectively. The genome of *D. acetexigens* consists of a single 3.68 Mbp circular chromosome with a G+C content of 60.3 %. In total, the genome encodes 3,466 genes, from which 3,386 are protein-coding genes, 51 tRNAs, 17 pseudogenes, and a duplication of the 16S-5S-23S rRNA operon. These genome statistics are similar to those of other *Desulfuromonas* species [23]. Only 54.5% of the protein-coding genes in *D. acetexigens* had KO identifiers/KEGG ortholog annotations. Analysis of the central metabolism pathways revealed that *D. acetexigens* possesses a vast genetic potential for the metabolism of carbon, nitrogen, sulfur, fatty acids, amino acids, cofactors, vitamins, and transport systems (see sections below), consistent with its ability to grow in diverse environmental conditions.

The proteome sequencing of *D. acetexigens* identified a total of 2135 proteins in cultures grown with fumarate as the electron acceptor (Supplementary Tables 1-3), representing 63.7% of all predicted proteins encoded in the *D. acetexigens* genome. A quantitative analysis of the identified proteins can be found in Supplementary Table 3. Approximately 62% of the identified proteins were associated with KEGG functional categories, while the remaining were not classified into known orthologous groups (Supplementary Table 5). The proteins with the highest levels of expression were associated with energy production and conversion (Supplementary Table 3). Ferrous iron transport proteins and ferritins were expressed at levels comparable to those related to energy production and conversion, which is not surprising given the large number of cytochromes present in *D. acetexigens*, and the fact that each heme group in these cytochromes contains an iron atom. The second-highest abundance proteins were associated with ribosomal structure, translational modification, biogenesis, protein turnover, and chaperones (Supplementary Table 3). The expression of proteins involved in central metabolic pathways was further analyzed for *D. acetexigens*. Complete metabolic pathways of central carbon metabolism (i.e., Glycolysis (Embden-Meyerhof pathway), gluconeogenesis, pyruvate oxidation, tricarboxylic acid (TCA) cycle, pentose phosphate pathway, phosphoribosyl diphosphate, UDP-N-acetyl-D-glucosamine biosynthesis), energy metabolism (i.e., carbon fixation, Phosphate acetyltransferase-acetate kinase pathway, ATP synthesis), lipid metabolism (i.e., fatty acid biosynthesis, beta-Oxidation, acyl-CoA synthesis), nucleotides, amino acids, glycan, cofactors, and vitamin metabolism were identified and are summarized in Supplementary Table 6.

Overall, the metabolic pathways described above and in the "Cytochrome diversity and electron transfer potential of *D. acetexigens*" section were found to be expressed in the *D. acetexigens* proteome. Interestingly, we detected the expression of the cytochrome TRO81949.1, which has a molecular weight of approximately 215 kDa (2081 aa) and a high number of predicted heme-binding motifs (86 hemes). To put this into perspective, the largest identified cytochrome monomer containing multiple

hemes is a hypothetical protein from *Agromonas oligotrophica* S58 that contains 85 CXXCH motifs [28, 29]. Currently, there are no structures available for cytochromes that contain more than 16 hemes per monomer [28]. Therefore, it is unclear what their function is or why it is necessary to generate a single protein with so many hemes. The high number of hemes may suggest a remarkable capability for electron storage and transfer [30].

Central carbon metabolism

Central metabolism of *D. acetexigens* includes a non-oxidative pentose phosphate pathway (TRO82226.1, TRO80616.1, TRO83176.1, TRO82810.1); glycolysis (Embden-Meyerhof pathway) (TRO83980.1, TRO79694.1, TRO83610.1, TRO80532.1, TRO83981.1, TRO79693.1, TRO82764.1, TRO83982.1, TRO79745.1, TRO83770.1, TRO79356.1, TRO84012.1, TRO82766.1, TRO82767.1); and gluconeogenesis (TRO81769.1, TRO78334.1, TRO81235.1, TRO79531.1). These pathways are linked by pyruvate:ferodoxin oxidoreductase (TRO83891.1, TRO83890.1, TRO80610.1) and a pyruvate dehydrogenase (TRO83323.1) to a complete tricarboxylic acid (TCA) cycle that includes the eukaryotic-like citrate synthase (TRO82332.1, TRO81252.1), characteristic of other *Geobacter* and *Desulfuromonas* strains [24, 31]. Central to the metabolism of *D. acetexigens* is the capability to anaerobically oxidize acetate to CO_2 using a variety of electron acceptors such as elemental sulfur, fumarate, malate, or electrode [8, 9, 21, 23]. The initial activation of acetate by acetate kinase (TRO79460.1, TRO79136.1) converts acetate to acetyl-phosphate, which acts as an intracellular signal for various phosphorylation-dependent signaling systems [32]. In the second reaction, phosphate acetyltransferase (TRO79138.1) converts acetyl-phosphate to acetyl-CoA, which is oxidized in the TCA cycle, with the products used to generate a proton gradient for ATP synthesis. The electrons released from these reactions can be transferred to insoluble external electron acceptors (e.g.,

electrode). It is important to mention that due to the reversibility of the reactions performed by the acetate kinase and the phosphate acetyltransferase, there is a possibility that *D. acetexigens* can also synthesize acetate under certain conditions. Even though *D. acetexigens* express all the set of enzymes required to metabolize sugars such as glucose, the lack of apparent transporters for sugar uptake (i.e., glucose/galactose transporter, Supplementary Table 1) highlights the importance of acetate metabolism to this organism. Interestingly, *D. acetexigens* expresses enzymes that might participate in the reductive acetyl-CoA pathway (Wood-Ljungdahl pathway) (i.e., TRO81902.1, TRO79141.1, TRO79142.1, TRO83183.1, TRO83182.1). This pathway can use one-carbon compounds and acetate as substrates for energy generation and carbon assimilation [33]. However, genes encoding formate-tetrahydrofolate ligase (*fhs*), 5-methyltetrahydrofolate corrinoid/iron sulfur protein methyltransferase (*acsE*), anaerobic carbon-monoxide dehydrogenase (*acsA*), and acetyl-coA synthase (*acsB*) are lacking in *D. acetexigens*. It has been reported that *G. sulfurreducens* also encodes genes that might participate in the acetyl-CoA pathway. However, unlike *D. acetexigens, G. sulfurreducens* possesses a gene encoding for carbon-monoxide dehydrogenase and lacks the gene encoding for formate-tetrahydrofolate ligase to complete the pathway [27].

Previous studies have reported that *Desulfuromonas* species can support growth from the oxidation of propionate, malate, lactate, and fumarate coupled with dissimilatory iron reduction [9, 34]. Therefore, other pathways of carbon metabolism that can serve as electron donor or carbon sources for *D. acetexigens* such as propionate, malate, and lactate, were also investigated. Analysis of the genome and proteome suggests that *D. acetexigens* encodes and expresses the enzymatic machinery to metabolize malate into oxaloacetate (malate dehydrogenase, TRO79526.1) and oxaloacetate into pyruvate (malate dehydrogenase (oxaloacetate-decarboxylating), TRO82345.1, TRO82771.1). In contrast, we did not find enzymes for the activation of propionate or metabolism of propionyl-CoA. However, as has been suggested for different species of *Geobacter*, propionate might be activated into

propionyl-CoA by the same enzymes that activate acetate [22, 35], which are expressed by *D. acetexigens*. Once propionyl-CoA is produced, it can be metabolized together with oxaloacetate into pyruvate by an oxaloacetate-decarboxylase (TRO82345.1, TRO82771.1). *D. acetexigens* does not have apparent enzymatic apparatus to use lactate as electron donor or carbon source, but it does have a nickel-dependent lactate racemase (TRO78339.1), which might be involved in the interconversion of the D- and L-enantiomers of lactic acid [36].

D. acetexigens utilizes fumarate as an electron acceptor in combination with acetate or other electron donors [21]. Additionally, it has been reported that some *Desulfuromonas* spp. can grow when provided with only fumarate, which can be fermented in the absence of acetate [9, 34]. The succinate dehydrogenase complex of *D. acetexigens* (TRO79507.1, TRO79506.1) also functions as a respiratory fumarate reductase in association with a *b*-type cytochrome [20]. Some *Geobacter* species such as *G. metallireducens*, *G. anodireducens* and *G. soli* are incapable of growth with fumarate as an electron acceptor unless they are transformed with a plasmid that expresses the dicarboxylic acid exchange transporter gene *dcuB* [20]. *D. acetexigens* expresses the C4-dicarboxylate transporter (TRO81908.1) which is required for the transport of aspartate, malate, fumarate, and succinate by many bacterial species [20]. The expression of the transporter correlates with the capability of *D. acetexigens* to metabolize those compounds. It is known that *D. acetexigens* can use malate as an electron acceptor [21]. Our analysis revealed that *D. acetexigens* has the potential to produce fumarate from malate by the action of fumarate hydratase (TRO83681.1, TRO82246.1).

Nitrogen metabolism

A complete set of genes for nitrogen fixation to ammonia (*nifDKH*, TRO83952.1, TRO83953.1, TRO83954.1) and the dinitrogenase iron-molybdenum cofactor biosynthesis proteins (TRO83962.1,

TRO83961.1, TRO81896.1, TRO80658.1, TRO80659.1) are expressed by *D. acetexigens*. It is common that members of the family *Geobacteraceae* contain genes for nitrogen fixation, which explains their ability to compete in nitrogen-limited subsurface environments [37]. Nitrate transporters and nitrate reductase genes are absent in *D. acetexigens*, excluding the possibility of dissimilatory nitrate reduction to ammonium (DNRA) and the use of nitrate as electron acceptor. However, *D. acetexigens* has a defined nitrite metabolism including an ammonia-forming cytochrome *c* nitrite reductase (TRO83645.1, TRO77878.1, TRO83644.1), a formate/nitrite transporter family protein (TRO83729.1), hydroxylamine reductases that can metabolize hydroxylamine into nitrite (TRO79796.1, TRO84146.1) and a nitronate monooxygenase (TRO80278.1) that can produce nitrite from nitroalkanes. The ammonia produced by nitrogen fixation or dissimilatory reduction of nitrite can be assimilated into the central metabolism of *D. acetexigens* by the action of glutamine synthetase (TRO82773.1) and glutamate dehydrogenase (TRO79145.1).

Sulfur metabolism

Our analysis disclosed the expression of enzymes for assimilatory sulfate reduction in *D. acetexigens*. Sulfate is first converted into adenylyl sulfate (APS) and 3'-phosphoadenylyl sulfate (PAPS) by the bifunctional enzyme sulfate adenyltransferase (CysNC, CysD; TRO83236.1, TRO83235.1). PAPS is further metabolized into sulfite by the phosphoadenosine phosphosulfate reductase (CysH, TRO83234.1) and finally reduced into sulfide by a sulfide reductase (Sir, TRO83237.1). Our analysis also revealed the expression of polysulfide reductases (TRO82651.1, TRO83143.1) and heterodisulfide reductases (TRO82275.1, TRO78595.1, TRO78596.1, TRO78608.1, TRO78598.1), which might be involved in different cellular processes of energy conservation [38, 39].

Desulfuromonas species are well known for their capability to perform dissimilatory sulfur reduction [21, 34, 40]. Despite the fact that *D. acetexigens* was reported to use sulfate as an electron acceptor for growth [21], the enzymes involved in the canonical microbial pathway for dissimilatory sulfate reduction such as sulfate adenylyltransferase (Sat), adenylyl-sulfate reductase (AprBA), and dissimilatory sulfite reductases (DsrAB) [41] were not found in *D. acetexigens* genome. These results suggest that the mechanism or proteins that *D. acetexigens* might use for dissimilatory sulfate reduction could be different from those reported in the literature [21]. Similarly, the exact molecular mechanism that other species from the *Desulfuromonas* genus employ to reduce elemental sulfur or polysulfides are still not fully understood and require further studies.

Fatty acid metabolism

D. acetexigens genome expresses the complete set of enzymes for the initiation (TRO82629.1, TRO81832.1, TRO80644.1, TRO79137.1, TRO84110.1, TRO84026.1, TRO83301.1, TRO84111.1) and elongation (TRO82628.1, TRO81878.1, TRO81125.1, TRO81879.1, TRO82753.1, TRO81889.1, TRO82626.1, TRO82000.1, TRO81840.1, TRO81881.1, TRO81891.1) of fatty acids. Additionally, it has a variety of enzymes for acyl-CoA metabolism (Supplementary Table 1, i.e., TRO83861.1, TRO83647.1, TRO84051.1, TRO83789.1, TRO82511.1, TRO81890.1). This multiplicity of genes for fatty acid metabolism suggests that *D. acetexigens* may also be able to utilize longer fatty acids as a source of carbon and/or electrons. Other species of *Desulfuromonas* have been reported to possess a high number of genes encoding for fatty acid degradation enzymes [42] and to be able to oxidize long-chain fatty acids coupled to Fe(III) reduction [40].

Amino acid biosynthesis and metabolism of cofactors and vitamins

D. acetexigens appears to be prototrophic for amino acids, vitamins, and cofactors. It expresses the full set of genes for the biosynthesis of cysteine (TRO83684.1, TRO83238.1, TRO82348.1), methionine (TRO82616.1, TRO83128.1, TRO81137.1, TRO82427.1, TRO83241.1, TRO83319.1, TRO83239.1, TRO83240.1, TRO83179.1), valine/isoleucine (TRO83994.1, TRO79339.1, TRO83996.1, TRO82333.1, TRO83995.1, TRO83997.1), isoleucine (TRO83994.1, TRO79339.1, TRO83996.1, TRO82333.1, TRO83995.1, TRO83997.1, TRO83682.1), leucine (TRO81138.1, TRO83688.1, TRO83320.1, TRO83689.1, TRO83690.1), lysine (TRO83128.1, TRO81137.1, TRO78829.1, TRO83319.1, TRO82335.1, TRO78828.1, TRO81799.1, TRO78831.1), threonine (TRO81137.1, TRO83128.1), ornithine (TRO83713.1, TRO79681.1, TRO79679.1, TRO78821.1, TRO78818.1, TRO81861.1), arginine (TRO78822.1, TRO78826.1, TRO78824.1), proline (TRO79350.1, TRO82446.1, TRO79349.1), tryptophan (TRO83879.1, TRO83295.1, TRO83878.1, TRO83296.1, TRO83293.1, TRO83294.1, TRO79088.1, TRO79087.1, TRO83877.1, TRO83298.1, TRO83297.1), and the Shikimate pathway for aromatic amino acid metabolism (TRO82791.1, TRO83733.1, TRO84116.1, TRO79705.1, TRO81270.1, TRO84115.1, TRO84117.1, TRO84156.1, TRO82221.1, TRO78561.1). Additionally, *D. acetexigens* is capable of full biosynthesis and metabolism of cofactors and vitamins such as the thiamine salvage pathway (TRO80246.1, TRO79092.1, TRO79794.1), NAD biosynthesis (TRO82583.1, TRO83633.1, TRO79351.1, TRO78950.1, TRO81982.1), Coenzyme A biosynthesis (TRO78571.1, TRO83911.1, TRO83635.1, TRO81165.1), pimeloyl-ACP biosynthesis (TRO82628.1, TRO81878.1, TRO81125.1, TRO81879.1, TRO79544.1, TRO79454.1, TRO82753.1, TRO81889.1, TRO82626.1, TRO82000.1, TRO81840.1, TRO81881.1, TRO81891.1), siroheme biosynthesis (TRO81813.1, TRO81809.1, TRO82220.1, TRO81997.1, TRO83155.1, TRO81810.1, TRO81807.1), the anaerobic uroporphyrinogen III and cobyrinate a,c-diamide pathways for cobalamin biosynthesis (TRO83156.1, TRO77783.1, TRO83157.1, TRO77787.1, TRO77841.1, TRO77839.1, TRO77785.1, TRO77784.1, TRO77788.1, TRO77792.1, TRO82644.1, TRO77786.1, TRO77840.1,

TRO81810.1, TRO81807.1, TRO77834.1, TRO77832.1, TRO83158.1, TRO77837.1, TRO77833.1, TRO77836.1, TRO83153.1, TRO77835.1, TRO82691.1, TRO77838.1), menaquinone biosynthesis (TRO84107.1, TRO82238.1, TRO80614.1, TRO82239.1, TRO80612.1, TRO82524.1), biotin biosynthesis (TRO84147.1, TRO83901.1, TRO79456.1, TRO83902.1), and heme biosynthesis (TRO80264.1, TRO80275.1, TRO81813.1, TRO81809.1, TRO80364.1, TRO82220.1, TRO83854.1, TRO81997.1, TRO81810.1). Overall, *D. acetexigens* possesses a vast potential for amino acids, cofactors, and vitamins biosynthesis. This enzymatic machinery may offer an adaptive advantage in substrate-limited environments.

Transport systems

Similar to other members of *Geobacteraceae*, the ATP-binding cassette (ABC) transport system for phosphate in *D. acetexigens* consist of a periplasmic phosphate-binding protein (PstS, TRO83053), two membrane proteins (PstC and PstA; TRO83052.1, TRO83051.1), and an ATP-binding protein (PstB, TRO83050.1), regulated by the phosphate signaling complex protein PhoU (TRO83049.1) [22]. Molybdate is transported in *D. acetexigens* by a high-affinity transport system composed of a periplasmic binding protein (ModA, TRO83957.1, TRO82273.1, TRO81199.1), an integral membrane protein (ModB, TRO83958.1, TRO81198.1), and an ATP-binding protein (ModC, TRO83959.1, TRO81197.1). The expression of multiple copies of the molybdate ABC transport system may suggest the importance of this mineral for *D. acetexigens* growth and metabolism. It is well known that molybdenum is present in the metallocluster of the catalytic site of the nitrogenase, where nitrogen gas is bound and reduced into ammonia [43]. This is why it is common to find the ABC transport cassette for molybdenum in nitrogen fixing bacteria [44]. *D. acetexigens* also expresses complete transport systems of tungstate (TRO79296.1, TRO79297.1, TRO79298.1), cobalt/nickel (TRO83163.1,

TRO82421.1, TRO83162.1), glycine/proline (TRO83115.1, TRO83114.1, TRO83113.1), iron (TRO81175.1, TRO81176.1, TRO81177.1), cobalt/zinc/cadmium (TRO78508.1, TRO83641.1, TRO82468.1, TRO80581.1), and an extensive number of proteins involved in the transport of acetate, formate, ammonium, potassium, magnesium, phospholipids, phosphonate, lipopolysaccharides, lipoproteins, and biopolymers (Supplementary Table 1).

Short-Term transcriptomic response of *D. acetexigens* to electron acceptor deprivation

The short-term response to the absence of electron acceptor availability was also evaluated in *D. acetexigens* biofilms (Supplementary Table 10). When *D. acetexigens* growth was switched from set potential to open-circuit voltage (OCV) mode, the most upregulated genes were related to flagellar biosynthesis, chemotaxis, and cytochrome biogenesis proteins (Supplementary Table 11). High expression of flagellar and chemotaxis proteins indicates that in the absence of electron acceptor, *D. acetexigens* may disassemble the biofilm and deploy motility complexes to search for alternative electron acceptors. The inner membrane-attached cytochrome biogenesis protein CcsB (TRO79786.1) which is essential for heme attachment in cytochrome biosynthesis [45], was highly upregulated together with a variety of periplasmic cytochromes with low (TRO79781.1, TRO82316.1, TRO83147.1, TRO82312.1, TRO78972.1) and high (TRO82434.1 (35 heme), TRO82434.1 (40 heme)) numbers of heme-binding motifs (Supplementary Table 11). Cytochrome *b* proteins (TRO79756.1, TRO82403.1) were also upregulated in the absence of electron acceptor, suggesting active electron transfer to the quinone pool (Supplementary Table 11). The upregulated low molecular weight periplasmic cytochromes might facilitate electron transfer between the quinone pool in the inner membrane and the outer membrane cytochromes.

It has been reported that redox molecules such as cytochromes can act as capacitors [46]. *G. sulfurreducens* cells can continue to metabolize acetate in the absence of an electron acceptor by overexpressing cytochromes and using them as capacitors to store up to 10^7 electrons per cell [47]. Therefore, the upregulation of genes involved in cytochromes biosynthesis in the absence of an electron acceptor suggests that *D. acetexigens* can still utilize its electron transfer ability and use cytochromes as temporary capacitors and short-term alternative of electron acceptor. Besides cytochromes, hydrogenases are another class of redox-active enzymes widely known for providing organisms with reducing power through hydrogen oxidation and for exhibiting reversible $2H^+/H_2$ electrocatalysis [48]. In addition to their catalytic function, hydrogenases can also act as electron sinks and, in some cases, serve as temporary redox capacitors, helping to buffer or store electrons during fluctuations in metabolic activity [48–51]. Our analysis revealed that a whole operon encoding a nickel-dependent hydrogenase large subunit (TRO82399.1), hydrogenase maturation protease (TRO82400.1), hydrogenase small subunit (TRO82401.1), hydrogenase protein HybA (TRO82402.1), and a Ni/Fe-hydrogenase cytochrome b subunit (TRO82403.1) was significantly expressed in the absence of an electron acceptor (Supplementary Table 11). Hydrogenases have been reported in other species of *Desulfuromonas* such as *D. acetoxidans* and *D. soudanensis* [24, 52]. Also, hydrogen (H_2) injection caused an increase in current in reactors with *D. soudanensis*, which is in agreement with the multiple Ni-Fe hydrogenases encoded in the genome of *D. soudanensis* [24]. In contrast, a recent study showed that *D. acetexigens* ceases current generation when H_2 is used as the sole electron donor [8]. However, our results suggest that *D. acetexigens* might employ hydrogenases to reduce protons and electrons to H_2, helping to turnover cellular electron carriers in the absence of an electron acceptor.

Supplementary References:

1. Kanehisa M, Sato Y, Morishima K. BlastKOALA and GhostKOALA: KEGG tools for functional characterization of genome and metagenome sequences. *J Mol Biol* 2016;**428**:726–31. https://doi.org/10.1016/j.jmb.2015.11.006
2. Allen JWA, Leach N, Ferguson SJ. The histidine of the c-type cytochrome CXXCH haem-binding motif is essential for haem attachment by the *Escherichia coli* cytochrome *c* maturation (Ccm) apparatus. *Biochem* J 2005;**389**:587–92. https://doi.org/10.1042/BJ20041894
3. Eren AM et al. Anvi'o: an advanced analysis and visualization platform for 'omics data. *PeerJ* 2015; **3**:e1319. https://doi.org/10.7717/peerj.1319
4. Delmont TO, Eren AM. Linking pangenomes and metagenomes: the Prochlorococcus metapangenome. *PeerJ* 2018;**6**:e4320. https://doi.org/10.7717/peerj.4320
5. Okabe S et al. Glycogen metabolism of the anammox bacterium *Candidatus* Brocadia sinica. *ISME J* 2021;**15**:1287–301. https://doi.org/10.1038/s41396-020-00850-5
6. Ishihama Y et al. Exponentially modified protein abundance index (emPAI) for estimation of absolute protein amount in proteomics by the number of sequenced peptides per protein. *Mol Cell Proteomics* 2005;**4**:1265–72. https://doi.org/10.1074/mcp.M500061-MCP200

7. Sapireddy V et al. Competition of two highly specialized and efficient acetoclastic electroactive bacteria for acetate in biofilm anode of microbial electrolysis cell. *npj Biofilms Microbiomes* 2021;**7**:47. https://doi.org/10.1038/s41522-021-00218-3

8. Katuri KP et al. Electroactive biofilms on surface functionalized anodes: The anode respiring behavior of a novel electroactive bacterium, *Desulfuromonas acetexigens*. *Water Res* 2020;**185**:116284. https://doi.org/10.1016/j.watres.2020.116284

9. Kuever J, Rainey FA, Widdel F. *Desulfuromonas*. *Bergey's Manual of Systematics of Archaea and Bacteria* 2015;1–7. https://doi.org/10.1002/9781118960608.gbm01039

10. Katuri KP, Albertsen M, Saikaly PE. Draft genome sequence of *Desulfuromonas acetexigens* strain 2873, a novel anode-respiring bacterium. *Genome Announc* 2017;**5**:e01522-16. https://doi.org/10.1128/genomeA.01522-16

11. Kim BC et al. Insights into genes involved in electricity generation in *Geobacter sulfurreducens* via whole genome microarray analysis of the OmcF-deficient mutant. *Bioelectrochemistry* 2008;**73**:70–5. https://doi.org/10.1016/j.bioelechem.2008.04.023

12. Strycharz SM et al. Gene expression and deletion analysis of mechanisms for electron transfer from electrodes to *Geobacter sulfurreducens*. *Bioelectrochemistry* 2011;**80**:142–50. https://doi.org/10.1016/j.bioelechem.2010.07.005

13. Logan BE et al. Electroactive microorganisms in bioelectrochemical systems. *Nat Rev Microbiol* 2019;**17**:307–19. https://doi.org/10.1038/s41579-019-0173-x

14. Snel B, Bork P, Huynen MA. Genome phylogeny based on gene content. *Nat Genet* 1999;**21**:108–10. https://doi.org/10.1038/5052

15. Dutilh BE et al. The consistent phylogenetic signal in genome trees revealed by reducing the impact of noise. *J Mol Evol* 2004;**58**:527–39. https://doi.org/10.1007/s00239-003-2575-6

16. Chen L-X et al. Accurate and complete genomes from metagenomes. *Genome Res* 2020;**30**:315–33. https://doi.org/10.1101/gr.258640.119

17. Campbell JH et al. UGA is an additional glycine codon in uncultured SR1 bacteria from the human microbiota. *Proc Natl Acad Sci* 2013;**110**:5540. https://doi.org/10.1073/pnas.1303090110

18. Heidelberg JF et al. Genome sequence of the dissimilatory metal ion–reducing bacterium *Shewanella oneidensis*. *Nat Biotechnol* 2002;**20**:1118–23. https://doi.org/10.1038/nbt749

19. Zhong C et al. Pan-genome analyses of 24 *Shewanella* strains re-emphasize the diversification of their functions yet evolutionary dynamics of metal-reducing pathway. *Biotechnol Biofuels* 2018;**11**:193. https://doi.org/10.1186/s13068-018-1201-1

20. Sun D et al. Characterization of the genome from *Geobacter anodireducens*, a strain with enhanced current production in bioelectrochemical systems. *RSC Adv* 2019;**9**:25890–9. https://doi.org/10.1039/C9RA02343G

21. Finster K, Bak F, Pfennig N. *Desulfuromonas acetexigens* sp. nov., a dissimilatory sulfur-reducing eubacterium from anoxic freshwater sediments. *Arch Microbiol* 1994;**161**:328–32. https://doi.org/10.1007/BF00303588

22. Aklujkar M et al. The genome of Geobacter bemidjiensis, exemplar for the subsurface clade of Geobacter species that predominate in Fe(III)-reducing subsurface environments. *BMC Genomics* 2010;**11**:490. https://doi.org/10.1186/1471-2164-11-490

23. Guo Y, Aoyagi T, Hori T. Comparative insights into genome signatures of ferric iron oxide- and anode-stimulated *Desulfuromonas* spp. strains. *BMC Genomics* 2021;**22**:475. https://doi.org/10.1186/s12864-021-07809-6

24. Badalamenti JP et al. Isolation and genomic characterization of *Desulfuromonas soudanensis* WTL, a metal- and electrode-respiring bacterium from anoxic deep subsurface brine. *Front Microbiol* 2016;**7**:913. https://doi.org/10.3389/fmicb.2016.00913

25. Mehta T et al. Outer membrane c-type cytochromes required for Fe(III) and Mn(IV) oxide reduction in *Geobacter sulfurreducens*. *App environ microbiol* 2005;**71**:8634–41. https://doi.org/10.1128/AEM.71.12.8634-8641.2005

26. Holmes DE et al. Microarray and genetic analysis of electron transfer to electrodes in *Geobacter sulfurreducens*. *Environ Microbiol* 2006;**8**:1805–15. https://doi.org/10.1111/j.1462-2920.2006.01065.x

27. Methé BA et al. Genome of *Geobacter sulfurreducens*: Metal Reduction in Subsurface Environments. *Science* 2003;**302**:1967–9. https://doi.org/10.1126/science.1088727

28. Edwards MJ et al. Role of multiheme cytochromes involved in extracellular anaerobic respiration in bacteria. *Protein Sci* 2020;**29**:830–42. https://doi.org/10.1002/pro.3787

29. He S et al. Comparative genomic analysis of neutrophilic iron(II) oxidizer genomes for candidate genes in extracellular electron transfer. *Front Microbiol* 2017;**8**:1584. https://doi.org/10.3389/fmicb.2017.01584

30. Akram M et al. A 192-heme electron transfer network in the hydrazine dehydrogenase complex. *Sci Adv* 2019;**5**:eaav4310. https://doi.org/10.1126/sciadv.aav4310

31. Bond D et al. Characterization of citrate synthase from *Geobacter sulfurreducens* and evidence for a family of citrate synthases similar to those of eukaryotes throughout the *Geobacteraceae*. *Appl Environ Microbiol* 2005;**71**:3858–65. https://doi.org/10.1128/AEM.71.7.3858-3865.2005

32. Wolfe AJ. The acetate switch. *Microbiol Mol Biol Rev* 2005;**69**:12–50. https://doi.org/10.1128/MMBR.69.1.12-50.2005

33. Ragsdale SW, Pierce E. Acetogenesis and the Wood–Ljungdahl pathway of CO_2 fixation. *Biochim Biophys Acta Proteins Proteom* 2008;**1784**:1873–98. https://doi.org/10.1016/j.bbapap.2008.08.012

34. An TT, Picardal FW. D*esulfuromonas carbonis* sp. nov., an Fe(III)-, S0 - and Mn(IV)-reducing bacterium isolated from an active coalbed methane gas well. *Int J Syst Evol Microbiol* 2015;**65**:1686–93. https://doi.org/10.1099/ijs.0.000159

35. Aklujkar M et al. The genome sequence of *Geobacter metallireducens*: Features of metabolism, physiology and regulation common and dissimilar to *Geobacter sulfurreducens*. *BMC Microbiology* 2009;**9**:109. https://doi.org/10.1186/1471-2180-9-109

36. Rankin JA et al. Lactate racemase nickel-pincer cofactor operates by a proton-coupled hydride transfer mechanism. *Biochemistry* 2018;**57**:3244–51. https://doi.org/10.1021/acs.biochem.8b00100

37. Holmes DE, Nevin KP, Lovley DR. Comparison of 16S rRNA, nifD, recA, gyrB, rpoB and fusA genes within the family Geobacteraceae fam. nov. *Int J Syst Evol Microbiol 2004;54:1591–9.* https://doi.org/10.1099/ijs.0.02958-0

38. Jormakka M et al. Molecular mechanism of energy conservation in polysulfide respiration. *Nat Struct Mol Biol* 2008;**15**:730–7. https://doi.org/10.1038/nsmb.1434

39. Kröninger L et al. Evidence for the involvement of two heterodisulfide reductases in the energy-conserving system of Methanomassiliicoccus luminyensis. *FEBS J* 2016;**283**:472–483. https://doi.org/10.1111/febs.13594

40. Coates JD et al. *Desulfuromonas palmitatis* sp. nov., a marine dissimilatory Fe(III) reducer that can oxidize long-chain fatty acids. *Arch Microbiol* 1995;**164**:406–13. https://doi.org/10.1007/BF02529738

41. Anantharaman K et al. Expanded diversity of microbial groups that shape the dissimilatory sulfur cycle. *ISME J* 2018;**12**:1715–28. https://doi.org/10.1038/s41396-018-0078-0

42. Yong G et al. Complete genome sequence of *Desulfuromonas sp*. strain AOP6, an iron(III) reducer isolated from subseafloor sediment. *Microbiol Resour Announc* 2021;**9**:e01325-19. https://doi.org/10.1128/MRA.01325-19

43. Hernandez JA, George SJ, Rubio LM. Molybdenum trafficking for nitrogen fixation. *Biochemistry* 2009;**48**:9711–21. https://doi.org/10.1021/bi901217p

44. Grunden AM, Shanmugam KT. Molybdate transport and regulation in bacteria. *Arch Microbiol* 1997;**168**:345–54. https://doi.org/10.1007/s002030050508

45. Kranz RG et al. Cytochrome *c* biogenesis: mechanisms for covalent modifications and trafficking of heme and for heme-iron redox control. *Microbiol Mol Biol Rev* 2009;**73**:510–528. https://doi.org/10.1128/MMBR.00001-09

46. Atkinson JT et al. Living electronics: A catalogue of engineered living electronic components. *Microb Biotechnol* 2023;**16**:507–33. https://doi.org/10.1111/1751-7915.14171

47. Esteve-Núñez A et al. Fluorescent properties of *c*-type cytochromes reveal their potential role as an extracytoplasmic electron sink in *Geobacter sulfurreducens*. *Environ Microbiol* 2008;**10**:497–505. https://doi.org/10.1111/j.1462-2920.2007.01470.x

48. Frey M. Hydrogenases: Hydrogen-activating enzymes. *ChemBioChem* 2002;**3**:153–160. https://doi.org/10.1002/1439-7633(20020301)3:2/3<153::AID-CBIC153>3.0.CO;2-B

49. Murphy BJ, Sargent F, Armstrong FA. Transforming an oxygen-tolerant [NiFe] uptake hydrogenase into a proficient, reversible hydrogen producer. *Energy Environ Sci* 2014;**7**:1426–1433. https://doi.org/10.1039/C3EE43652G

50. Furlan C et al. Structural insight on the mechanism of an electron-bifurcating [FeFe] hydrogenase. *eLife* 2022;**11**:e79361. https://doi.org/10.7554/eLife.79361

51. Vincent KA, Parkin A, Armstrong FA. Investigating and exploiting the electrocatalytic properties of hydrogenases. *Chem Rev* 2007;**107**:4366–413. https://doi.org/10.1021/cr050191u

52. Brugna M et al. First evidence for the presence of a hydrogenase in the sulfur-reducing bacterium *Desulfuromonas acetoxidans*. *J Bacteriol* 1999;**181**:5505–08. https://doi.org/10.1128/JB.181.17.5505-5508.1999

53. Jumper J et al. Highly accurate protein structure prediction with AlphaFold. *Nature* 2021;**596**:583–9. https://doi.org/10.1038/s41586-021-03819-2

Supplementary Figures

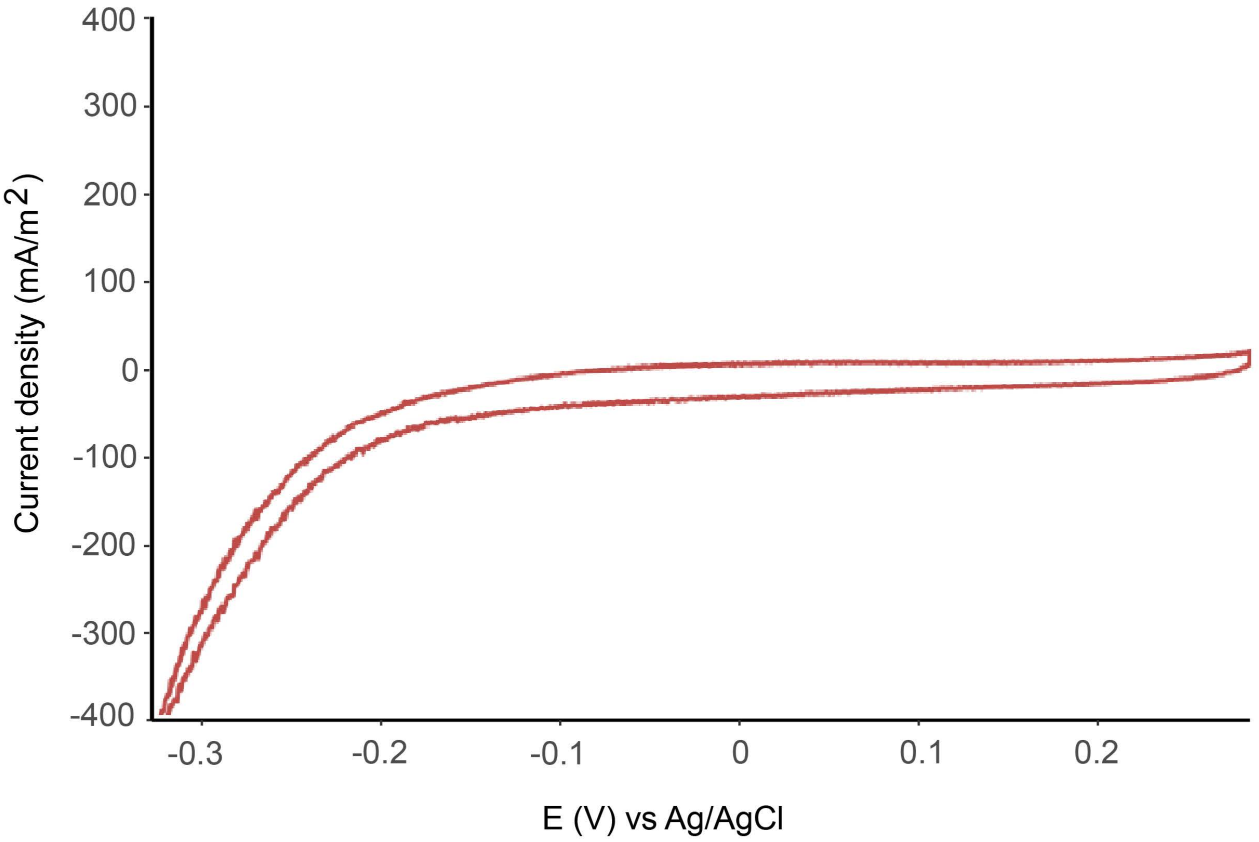

Supplementary Fig. 1 Cyclic voltammetry (CV) response (1 mV s^{-1} scan rate) of cell-free spent wash solution collected at the end of operation of MECs with *D. acetexigens* biofilm.

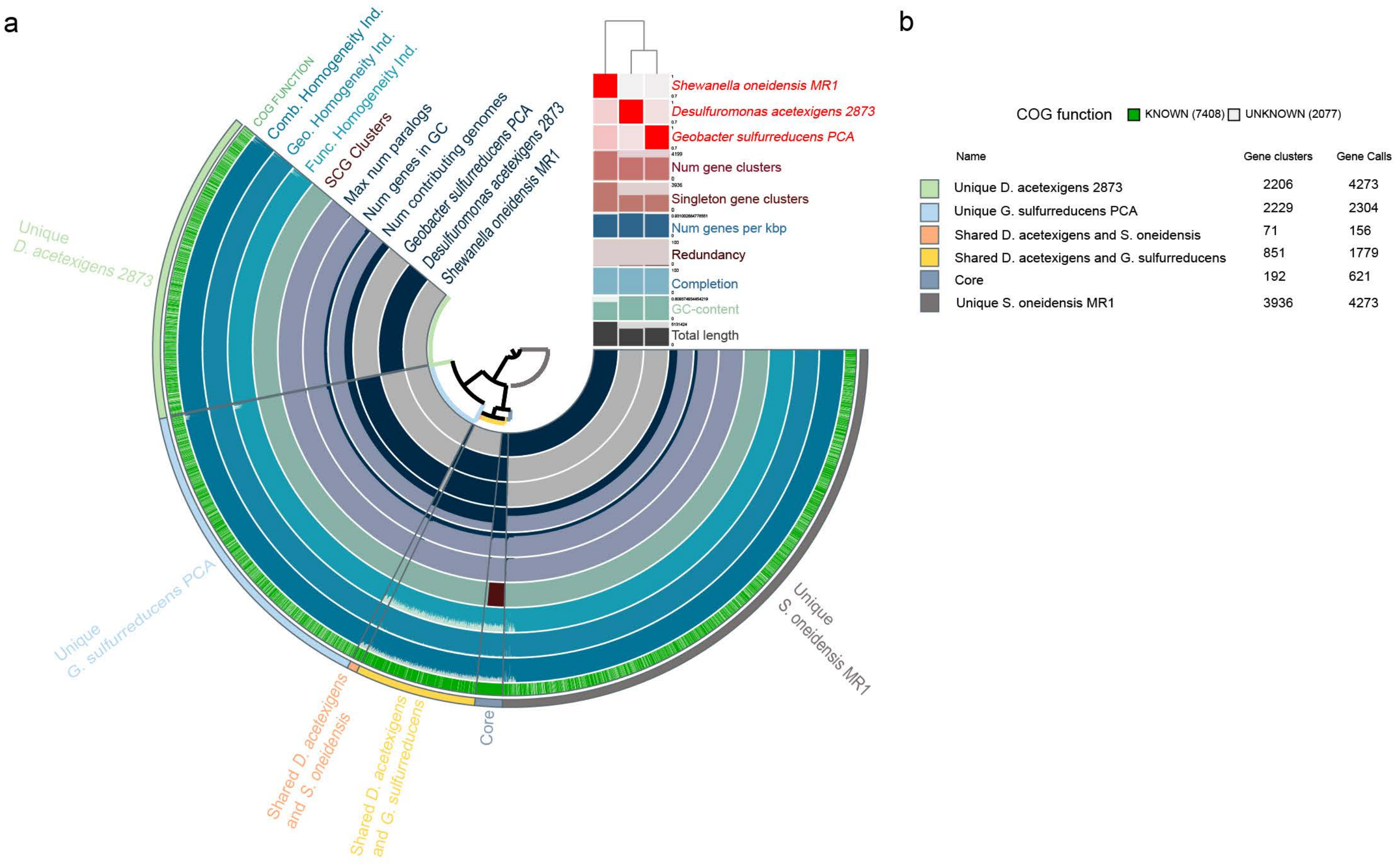

Supplementary Fig. 2 Pangenomic analysis of *D. acetexigens* 2873 (VJVV00000000.1), *G. sulfurreducens* PCA (NC_002939) and *S. oneidensis* MR-1 (GCA_000146165.2). a The radial layers indicate the occurrence of a gene cluster in a given genome as dark-colored bars, whereas light-colored bars indicate its absence. The first three layers (from inner to outer layer) of the radial plot represent individual genomes organized according to their phylogenetic relationship as indicated by the dendrogram. The next three layers "Number of contributing genomes", "Number of genes in gene cluster" and "Maximum number of paralogs" indicate the number of genomes containing each gene group (i.e., 1, 2 or 3 genomes), the number of genes in each gene cluster (bars are higher when a gene cluster is composed by more than one gene), and maximum number of genes contributed from a single genome (paralogs) per gene cluster, respectively. "SCG Cluster" layer shows single copy gene clusters.

The bar height of the layers "Func. Homogeneity Ind.", "Geo. Homogeneity Ind.", and "Comb. Homogeneity Ind" represent the functional homogeneity index (i.e., how conserved are the aligned amino acid residues across genes), geometric homogeneity index (i.e., how does the gap/residue distribution look like within a gene cluster regardless of the identity of amino acids), and the combined homogeneity index, respectively. "COG function" layer shows the gene groups in which at least one gene was functionally annotated using Pfams and/or clusters of orthologous groups of proteins (COGs). The outermost layer of the radial plot shows the unique, shared, and core gene clusters of the genomes. The size of the perimeter in the outermost layer is proportional to the total number of gene clusters. The phylogenetic relationship of the genomes is shown in the tree of the upper right part of the radial plot. Below the tree, from the top to bottom is shown a heatmap with the average nucleotide identity (ANI) between the genomes, a comparison between the number of gene clusters, number of singleton gene clusters, number of genes per Kbp, level of genomic redundancy, genome completion, GC content, and total genome length. **b** Each one of the total 9,485 gene clusters (7,408 with known COG function and 2,077 with unknown function) contains one or more genes contributed by one or more genomes. The table shows the number of unique, shared, and core gene and gene clusters of the genomes.

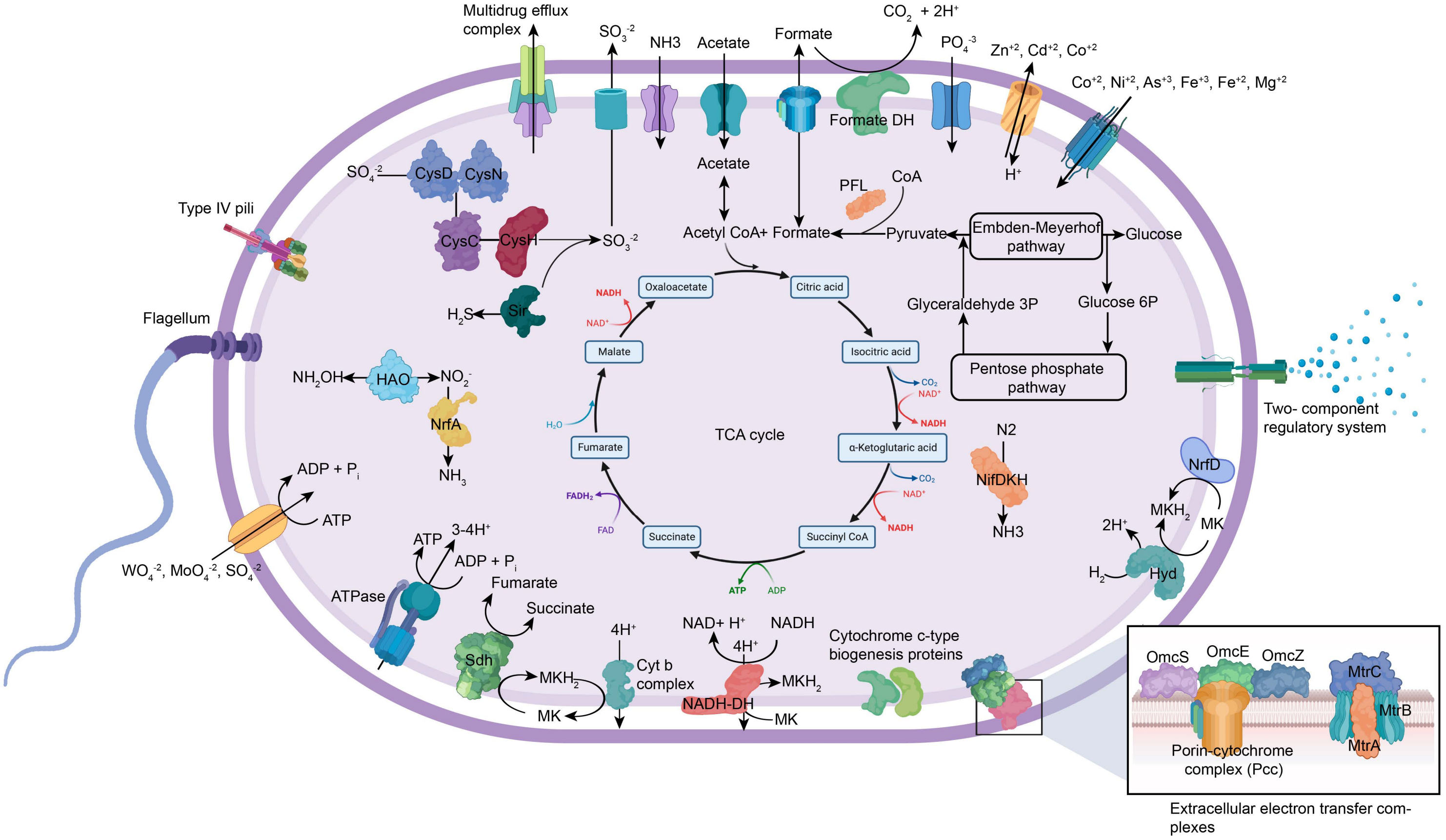

Supplementary Fig. 3 Metabolic reconstruction of *D. acetexigens*. Metabolic reconstruction for *D. acetexigens* was performed using KO identifiers/KEGG orthologs as reference. A metabolic pathway was considered to be present in *D. acetexigens* only if the complete set of enzymes necessary to perform a reaction or pathway was expressed at the protein level. Arrow lines indicate single or multiple steps in a pathway and the direction of the reaction. The full list of enzymes and metabolic pathways present in *D. acetexigens* can be found in Supplementary Tables 4-6.

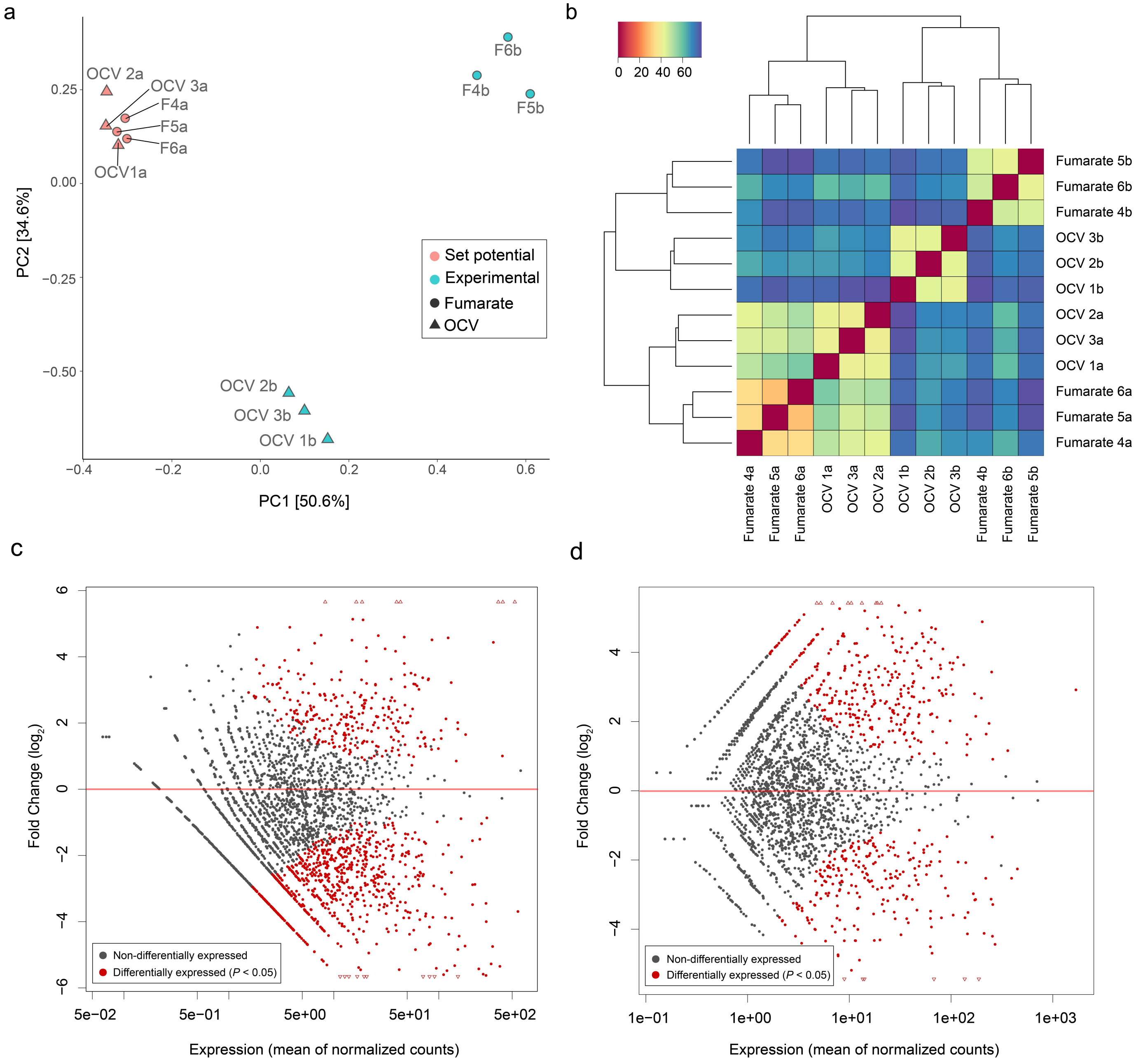

Supplementary Fig. 4 Overall transcriptomics similarity between biological replicate samples and differential expression analysis of *D. acetexigens* biofilm with respect to type (electrode vs fumarate) and availability of the external electron acceptor. **a** Principal component analysis (PCA) analysis of overall gene expression levels of triplicate reactors under set potential (electrode as the electron acceptor) and after switching to fumarate as the electron acceptor (soluble electron acceptor)

or open circuit voltage (OCV) mode. **b** Heatmap and hierarchical clustering of sample-to-sample distances. PCA analysis, heatmap and hierarchical clustering show high intra-replicate clustering and low inter-replicate clustering, indicating high similarity between biological replicates and suggesting differentially expressed genes across the experimental setups. MA plot of genes responding to the swish from set potential to fumarate as the electron acceptor (**c**) and set potential to OCV mode (**d**). Differentially expressed genes (DEG) ($P<0.05$) are shown in red. Non-differentially expressed genes are shown in grey.

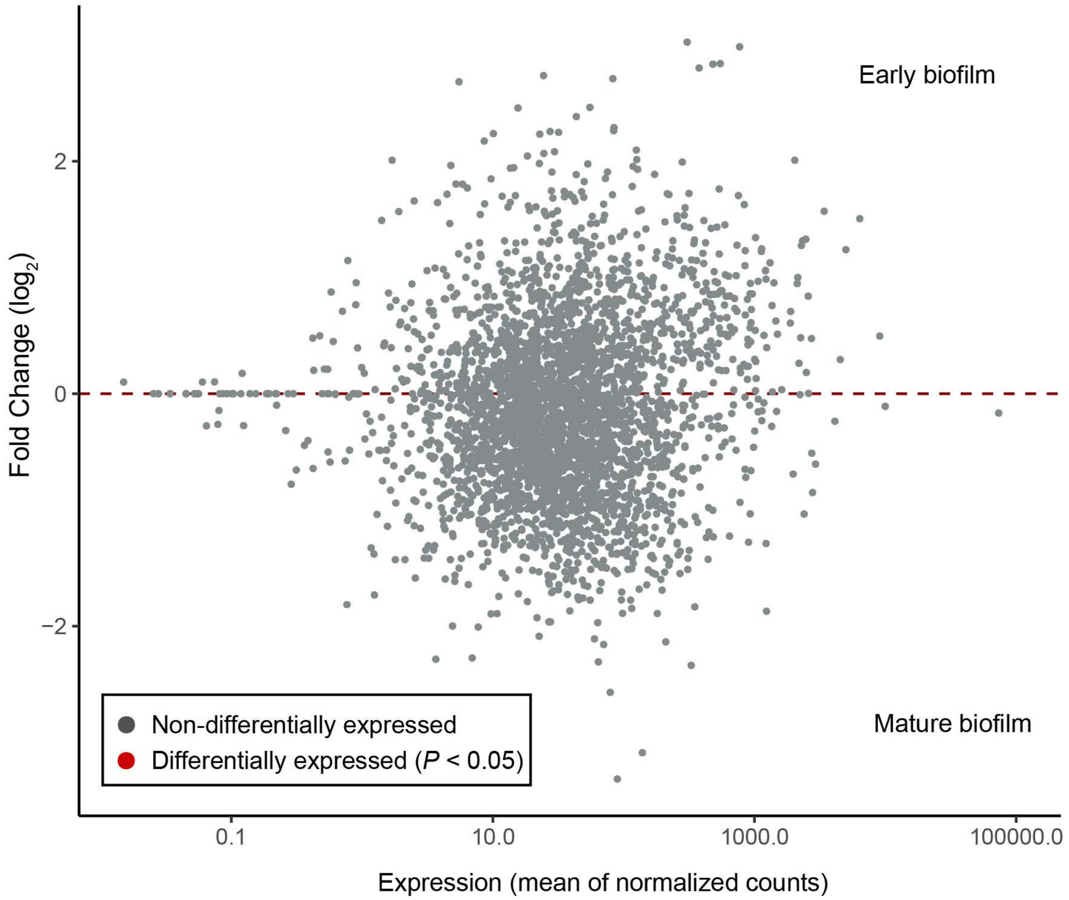

Supplementary Fig. 5 MA plot showing differential expression analysis between early and mature biofilm of *D. acetexigens*. Differentially expressed genes (DEGs) ($P<0.05$) are shown in red, while non-differentially expressed genes are shown in grey.

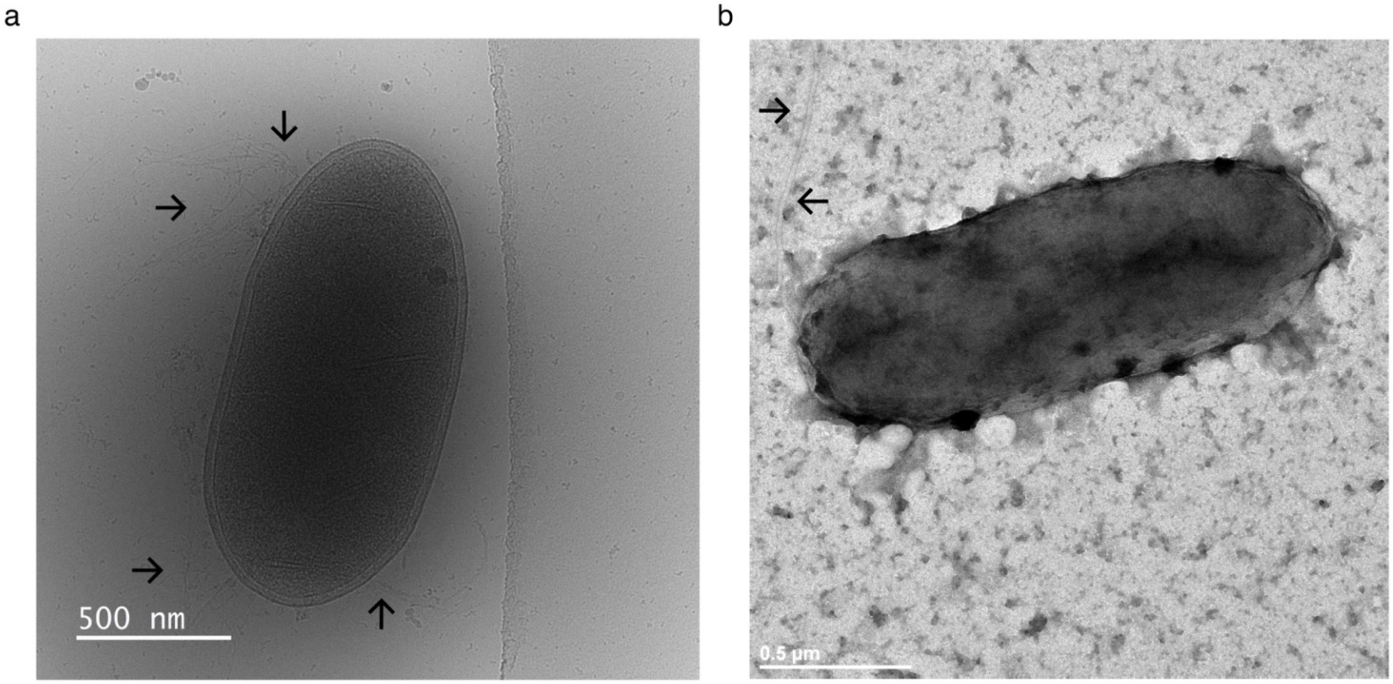

Supplementary Fig. 6 Transmission electron micrographs of *D. acetexigens*. Arrows in (**a**) and (**b**) indicate appendages and flagellum, respectively.

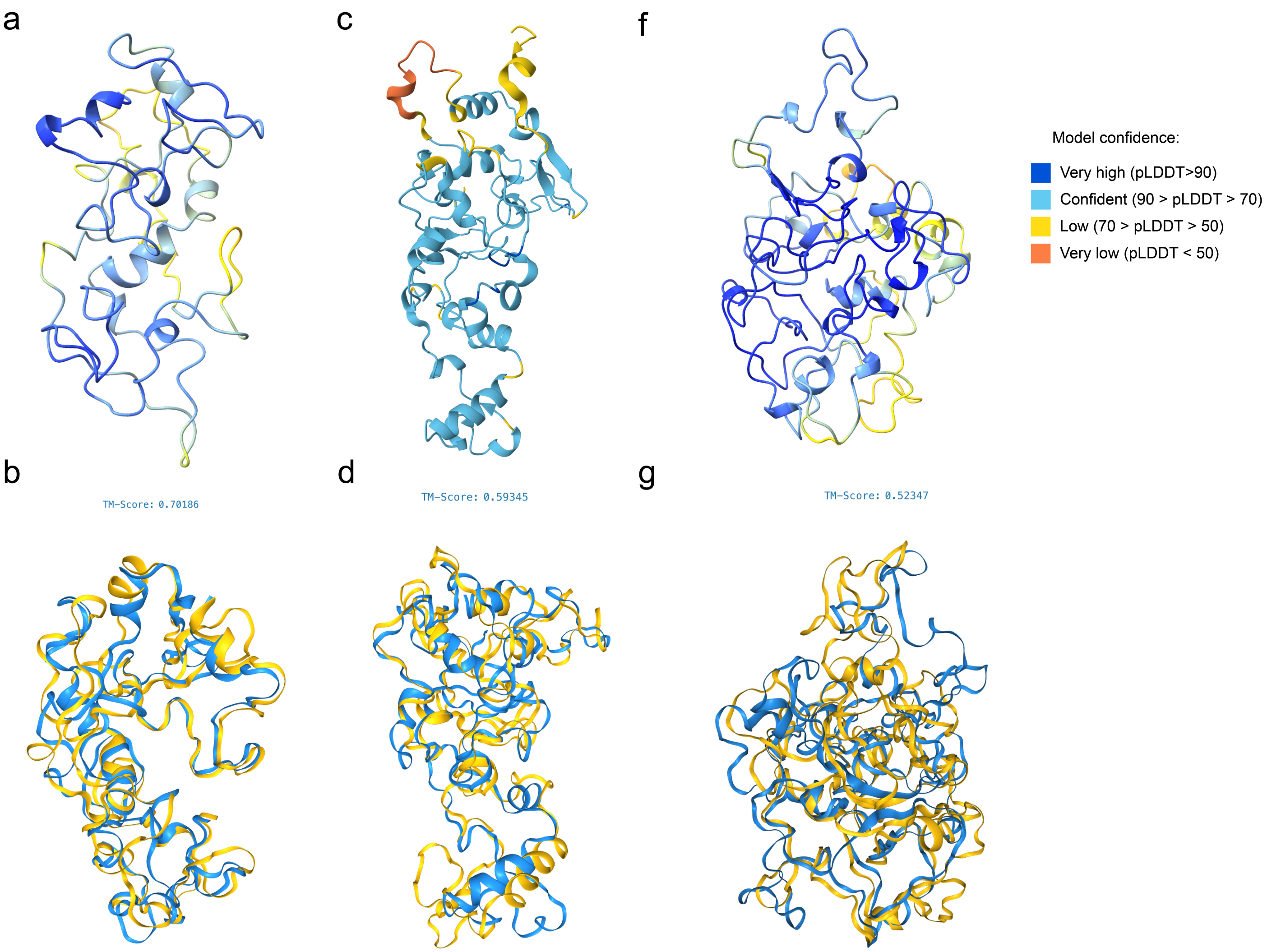

Supplementary Fig. 7 Structural prediction and structural alignment of Omc proteins of *D. acetexigens*. Predicted structural models for the OmcE, OmcZ, OmcS proteins were generated using AlphaFold [53] (a, c, f). The per-residue measure of local confidence (pLDDT) estimates how well the prediction would agree with experimentally determined structures [53]. Areas of high confidence appear in blue. Structural superposition alignments between *D. actexigens* OmcE, OmcZ, and OmcS and the best hit against the PDB database (b, d, g). The best hits correspond to the Cryo-EM resolved structures of OmcE (PDB 7TFS), OmcZ (PDB 8D9M), and OmcS (PDB 6EF8) nanowires from *Geobacter sulfurreducens*. The TM-Score indicates the metric (value between 0 and 1) for assessing the topological similarity between the evaluated protein structures, with 0.0 indicating no match, 1.0 being an exact match and >0.5 representing the same fold.

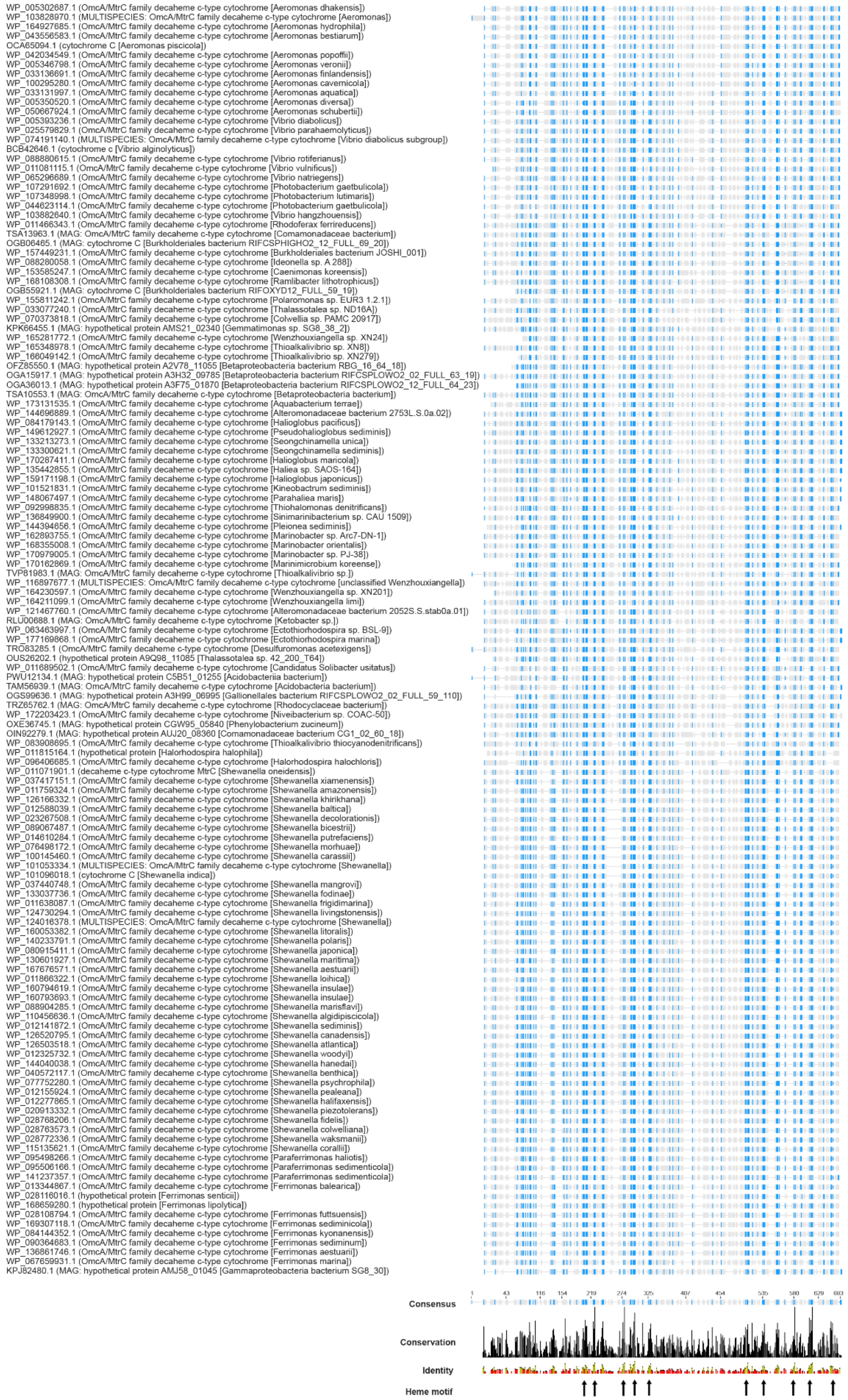

Supplementary Fig. 8 Sequence alignment of outer membrane OmcA/MtrC family decaheme *c*-

type cytochromes from various taxonomic groups. The accession number and name for each protein are provided at the beginning, followed by the name of each species in brackets. Residues with conservation >60% in the sequences appear in blue. Arrows indicate the heme binding sites, which correspond to conservation levels >90%.

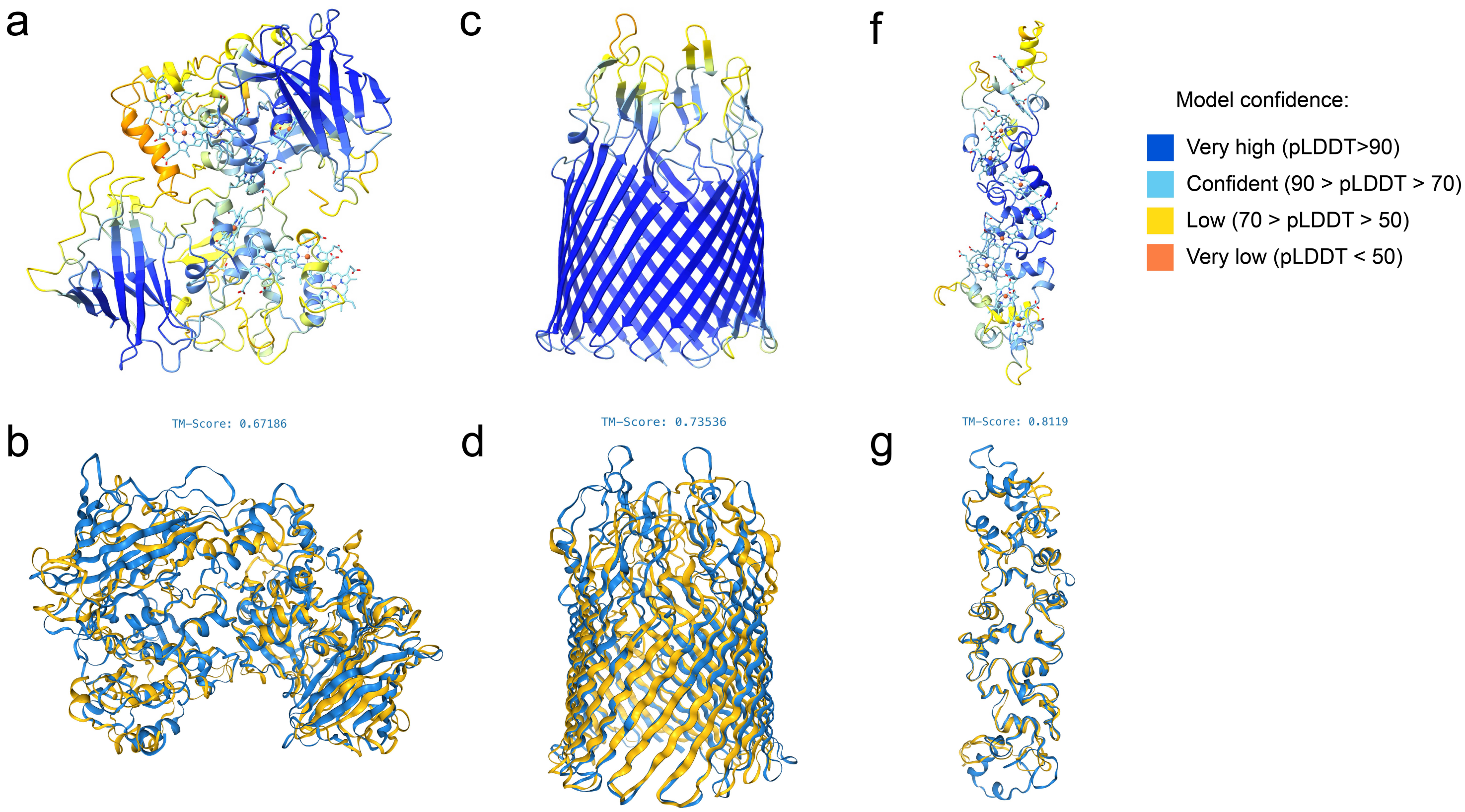

Supplementary Fig. 9 Structural prediction and structural alignment of Mtr proteins of *D. acetexigens*. Predicted structural models for the novel MtrC, MtrB, MtrA proteins were generated using AlphaFold [53] (a, c, f). The per-residue measure of local confidence (pLDDT) estimates how well the prediction would agree with experimentally determined structures [53]. Areas of high confidence appear in blue. Structural superposition alignments between *D. actexigens* MtrC, MtrB, and MtrA and the best hit against the PDB database (b, d, g). The best hit corresponds to the crystal structure of the Mtr complex of *Shewanella baltica* (PDB 6R2Q). The TM-Score indicates the metric (value between 0 and 1) for assessing the topological similarity between the evaluated protein structures, with 0.0 indicating no match, 1.0 being an exact match and >0.5 representing the same fold.

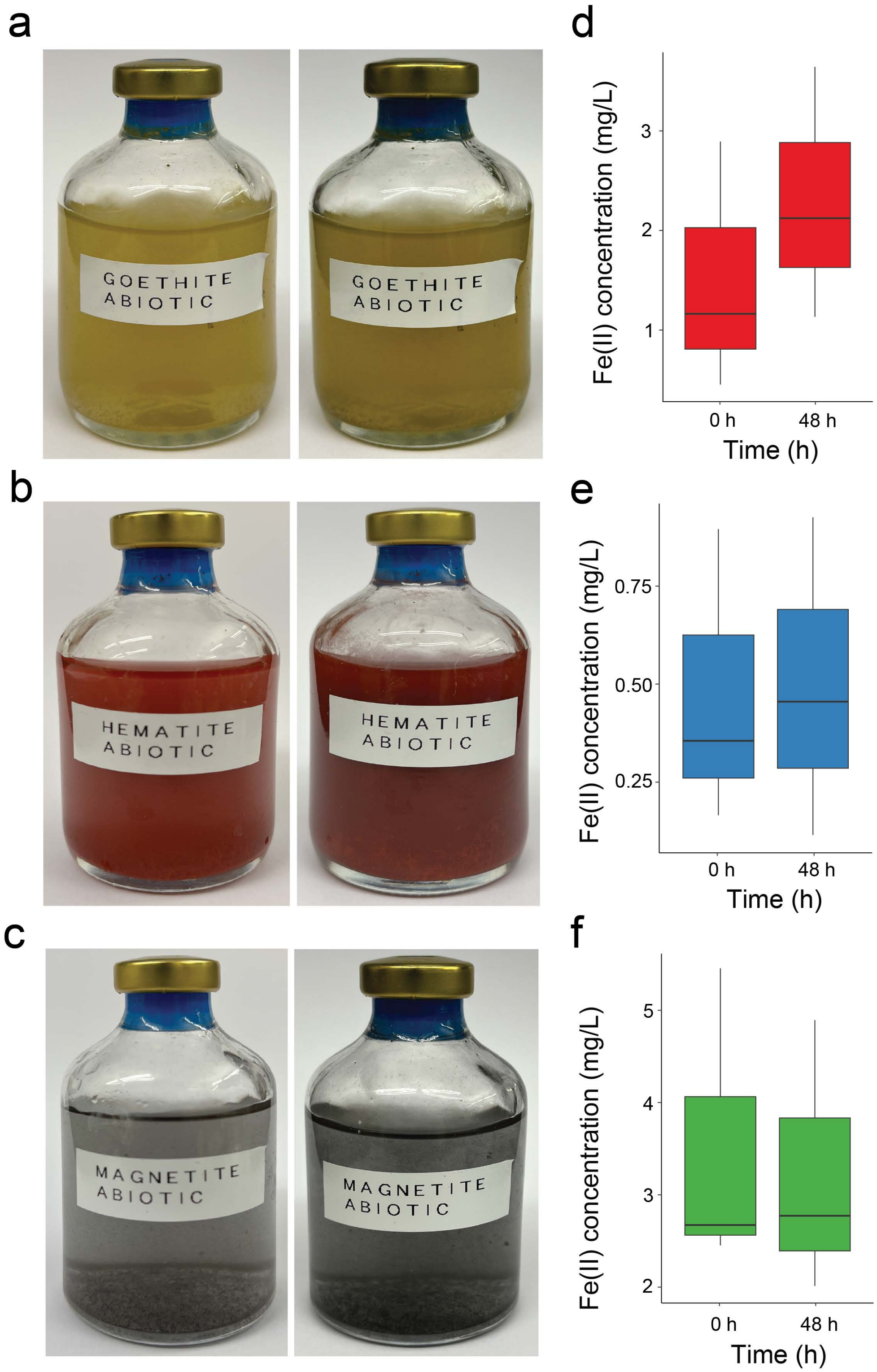

Supplementary Fig. 10 Abiotic incubations with iron oxides. Photographs of vials incubated with acetate as the electron donor and goethite (**a**), hematite (**b**), and magnetite (**c**) as the electron acceptors

at 0 h (left) and 48 h (right) after incubation. Iron (II) concentration in the medium at 0 h and 48 h after abiotic incubation with goethite (**d**), hematite (**e**), and magnetite (**f**).

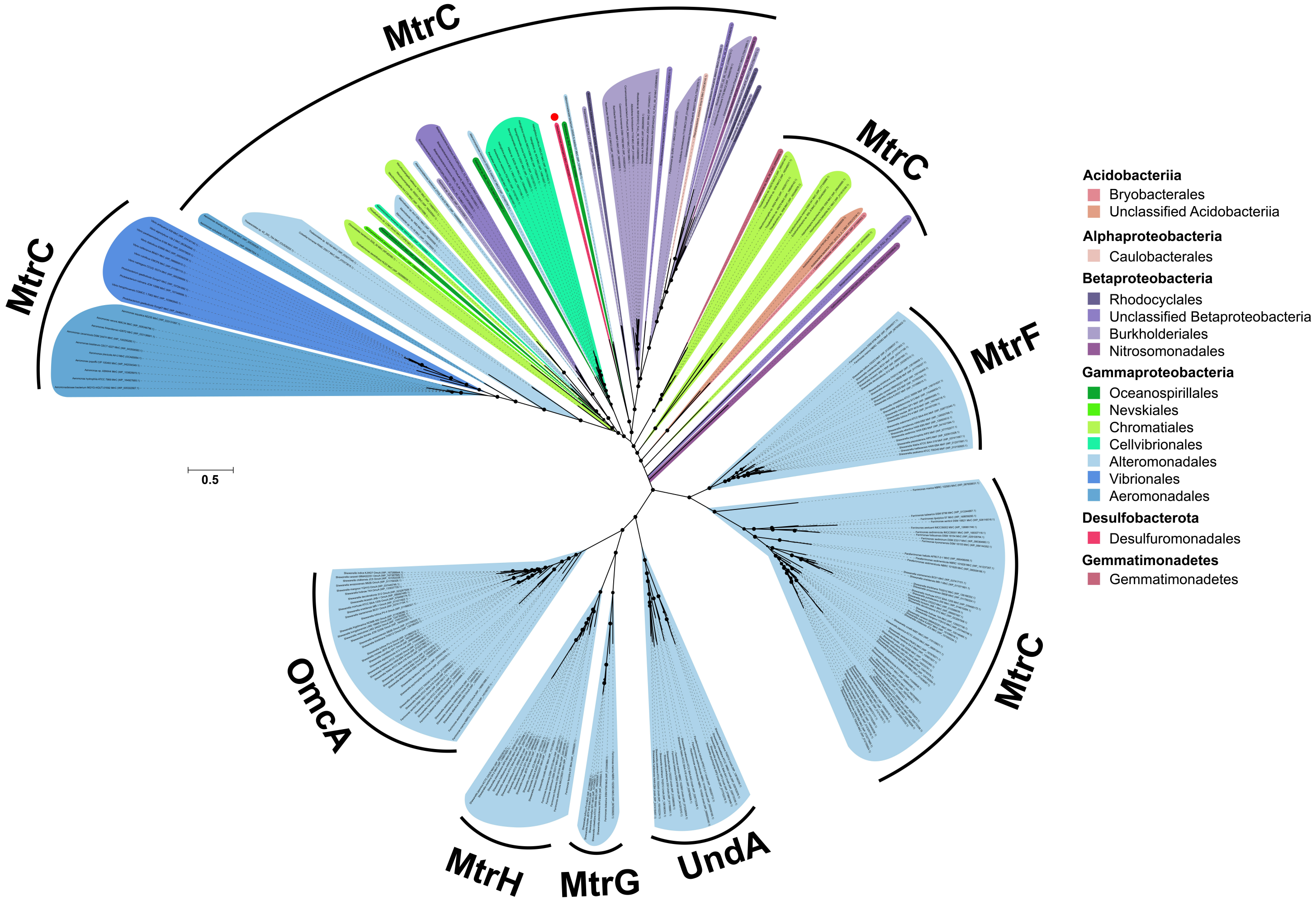

Supplementary Fig. 11 Phylogenetic relationship among known OmcA/MtrC decaheme *c*-type cytochromes from diverse bacteria and the novel *D. acetexigens* MtrC detected in this study. Maximum-likelihood phylogenetic tree constructed from a concatenated alignment of the known diversity of OmcA/MtrC decaheme *c*-type cytochromes and the novel *D. acetexigens* MtrC detected in this study. Color codes were assigned by taxonomic order. Bootstrap support values ≥90% from 1000 resamples are indicated with filled black dots. The scale bar represents average changes per amino acid position. MtrC, OmcA, MtrH, MtrG, UndA, and MtrF indicate members of the MtrC family of proteins. MtrC of *D. acetexigens* is indicated with a red dot.

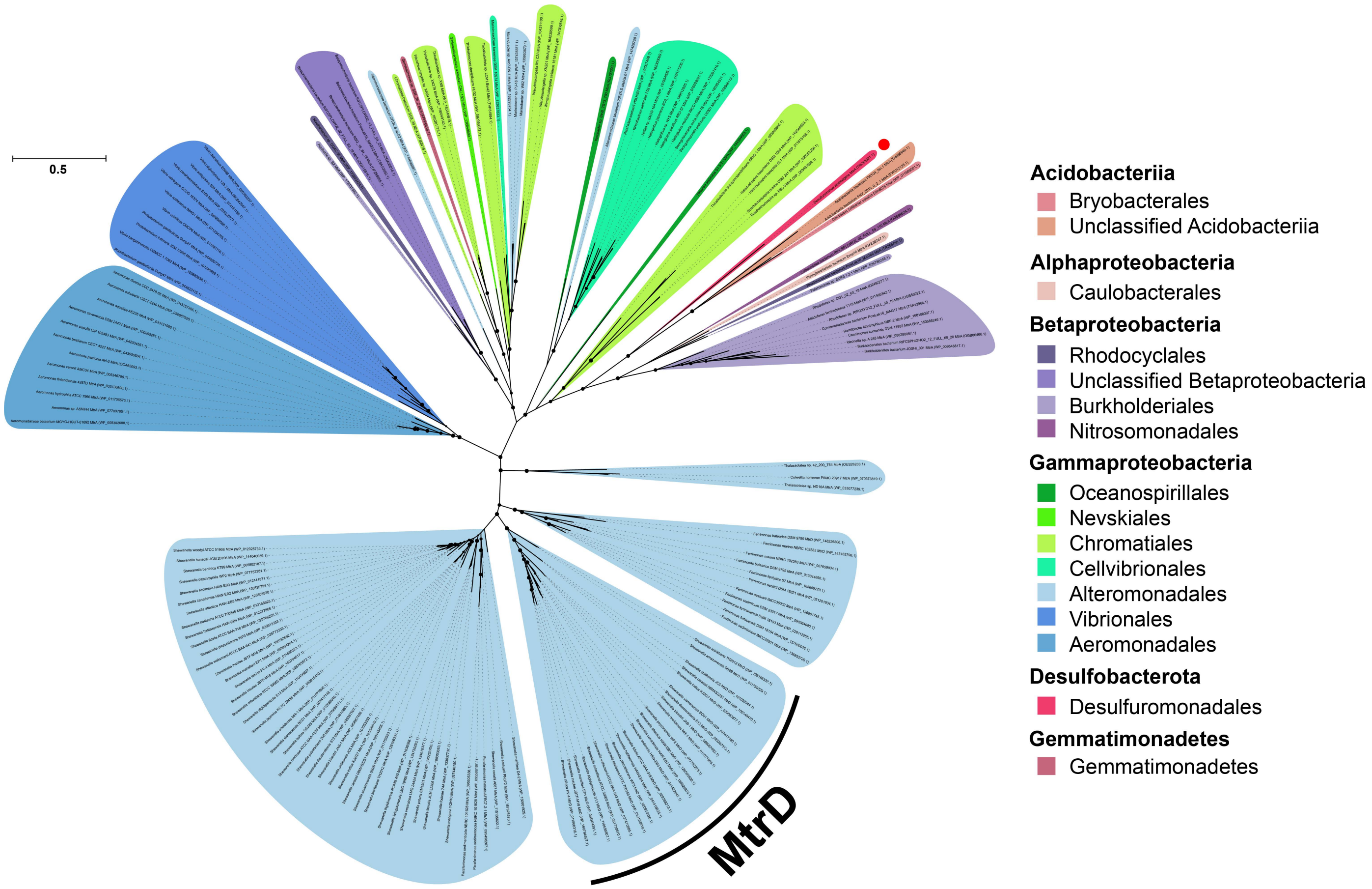

Supplementary Fig. 12 Phylogenetic relationship among known MtrA decaheme *c*-type cytochromes from diverse bacteria and the novel *D. acetexigens* MtrA detected in this study. Maximum-likelihood phylogenetic tree constructed from a concatenated alignment of the known diversity of MtrA decaheme *c*-type cytochromes and the novel *D. acetexigens* MtrA detected in this study. Color codes were assigned by taxonomic order. Bootstrap support values ≥90% from 1000 resamples are indicated with filled black dots. The scale bar represents average changes per amino acid position. MtrD indicates homologs of MtrA. MtrA of *D. acetexigens* is indicated with a red dot.

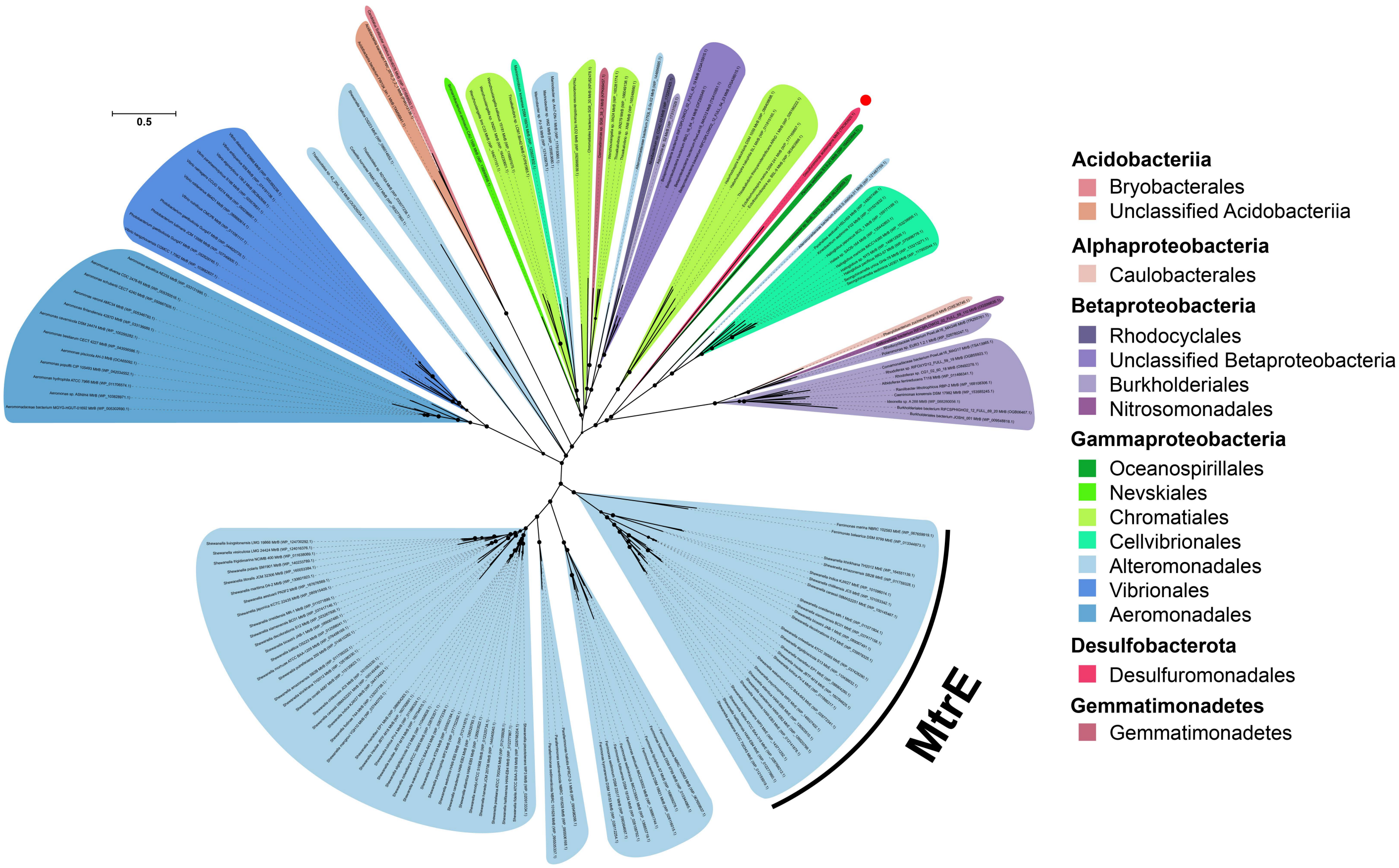

Supplementary Fig. 13 Phylogenetic relationship among known MtrB from diverse bacteria and the novel *D. acetexigens* MtrB detected in this study. Maximum-likelihood phylogenetic tree constructed from a concatenated alignment of the known diversity of MtrB β-barrel and the novel *D. acetexigens* MtrB detected in this study. Color codes were assigned by taxonomic order. Bootstrap support values ≥90% from 1000 resamples are indicated with filled black dots. The scale bar represents average changes per amino acid position. MtrE indicates homologs of MtrB. MtrB of *D. acetexigens* is indicated with a red dot.

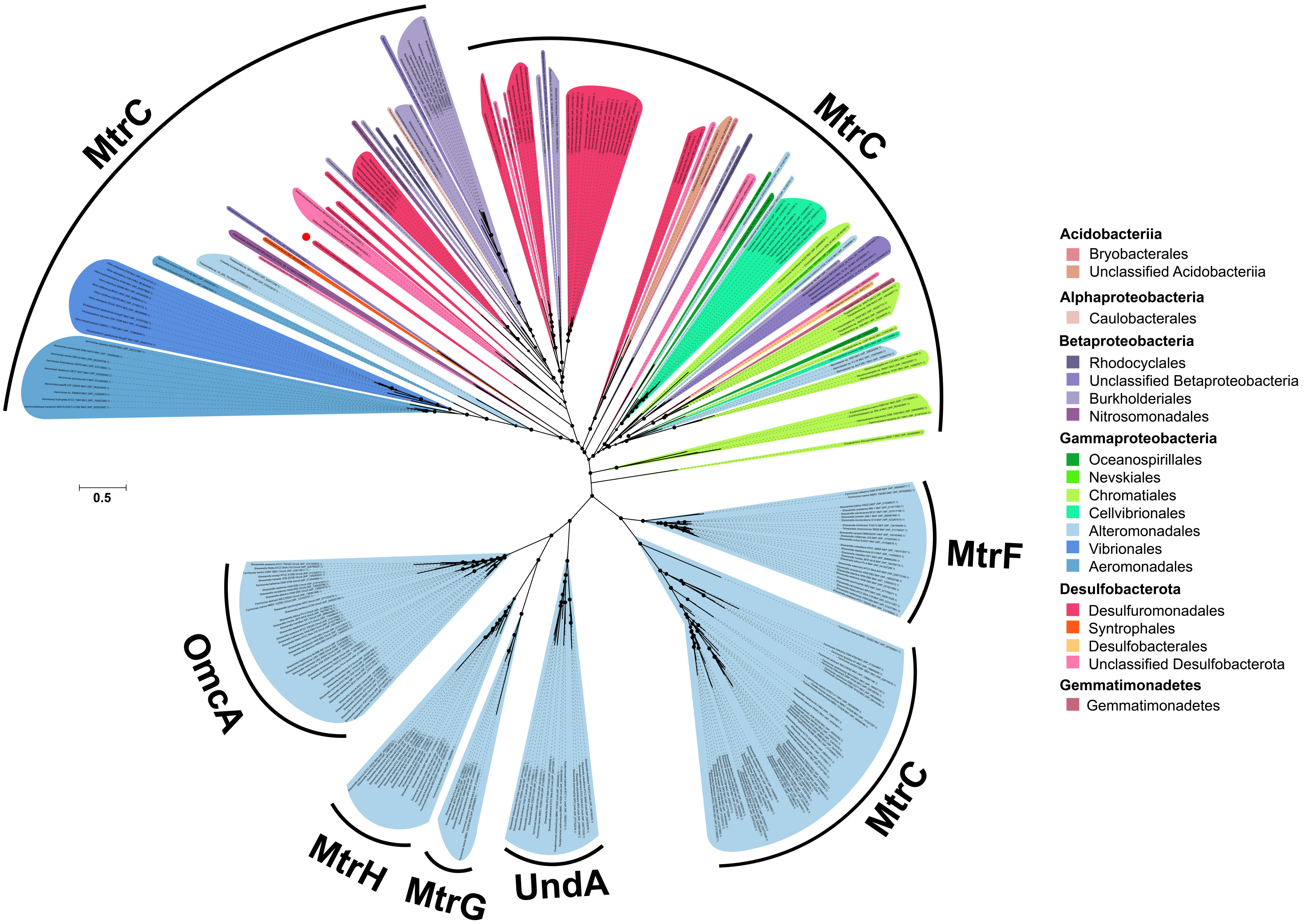

Supplementary Fig. 14 Phylogenetic relationship among known OmcA/MtrC decaheme *c*-type cytochromes from diverse bacteria and novel *Desulfobacterota* MtrC detected in this study. Maximum-likelihood phylogenetic tree constructed from a concatenated alignment of the known diversity of OmcA/MtrC decaheme *c*-type cytochromes and the novel *Desulfobacterota* MtrC detected in this study. Color codes were assigned by taxonomic order. Bootstrap support values ≥90% from 1000 resamples are indicated with filled black dots. The scale bar represents the average changes per amino acid position. MtrC, OmcA, MtrH, MtrG, UndA, and MtrF indicate members of the MtrC family of proteins. MtrC of *D. acetexigens* is indicated with a red dot.

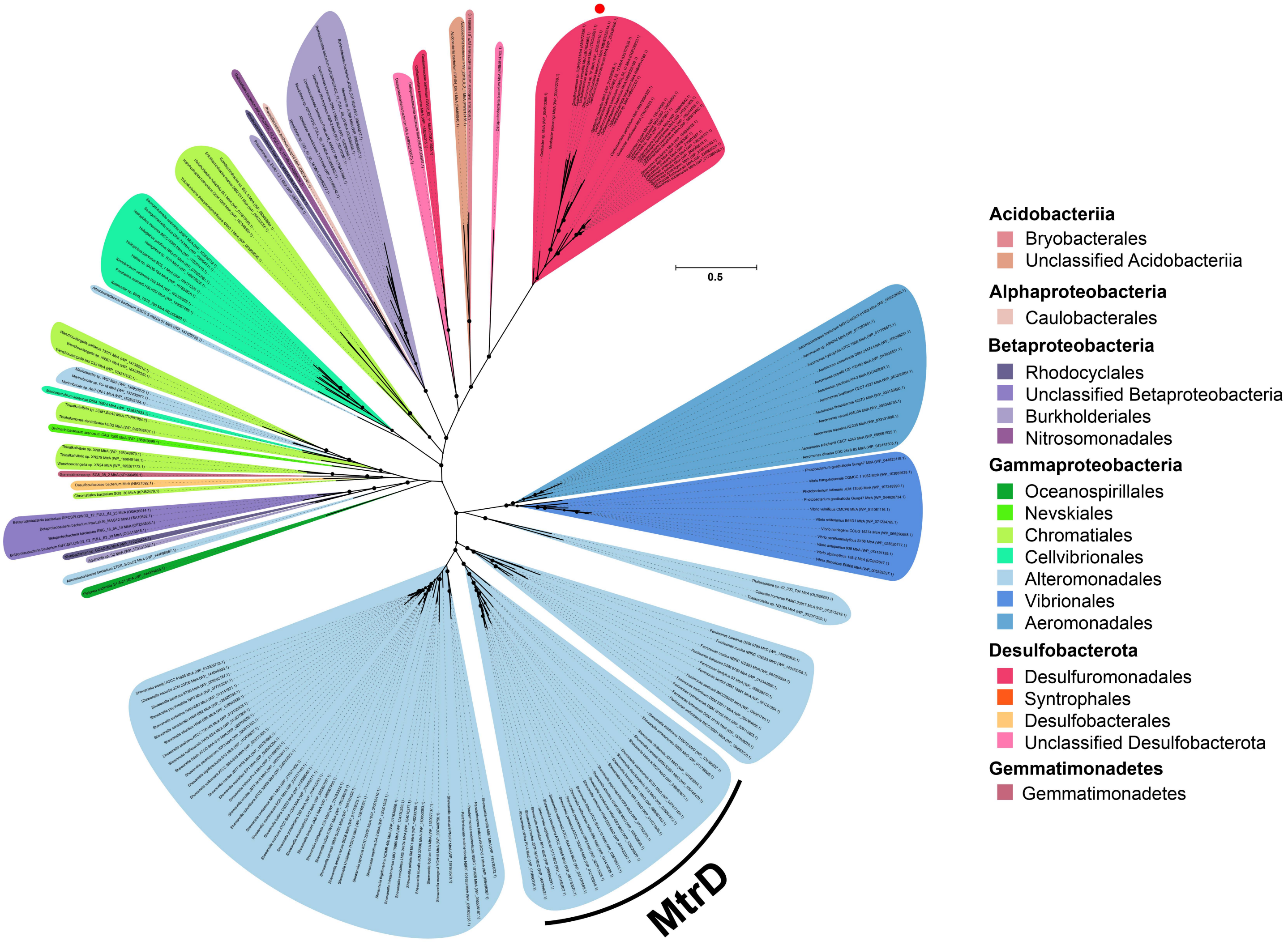

Supplementary Fig. 15 Phylogenetic relationship among known MtrA decaheme *c*-type cytochromes from diverse bacteria and the novel *Desulfobacterota* MtrA detected in this study. Maximum-likelihood phylogenetic tree constructed from a concatenated alignment of the known diversity of MtrA decaheme cytochromes and the novel *Desulfobacterota* MtrA detected in this study. Color codes were assigned by taxonomic order. Bootstrap support values ≥90% from 1000 resamples are indicated with filled black dots. The scale bar represents average changes per amino acid position. MtrD indicates homologs of MtrA. MtrA of *D. acetexigens* is indicated with a red dot.

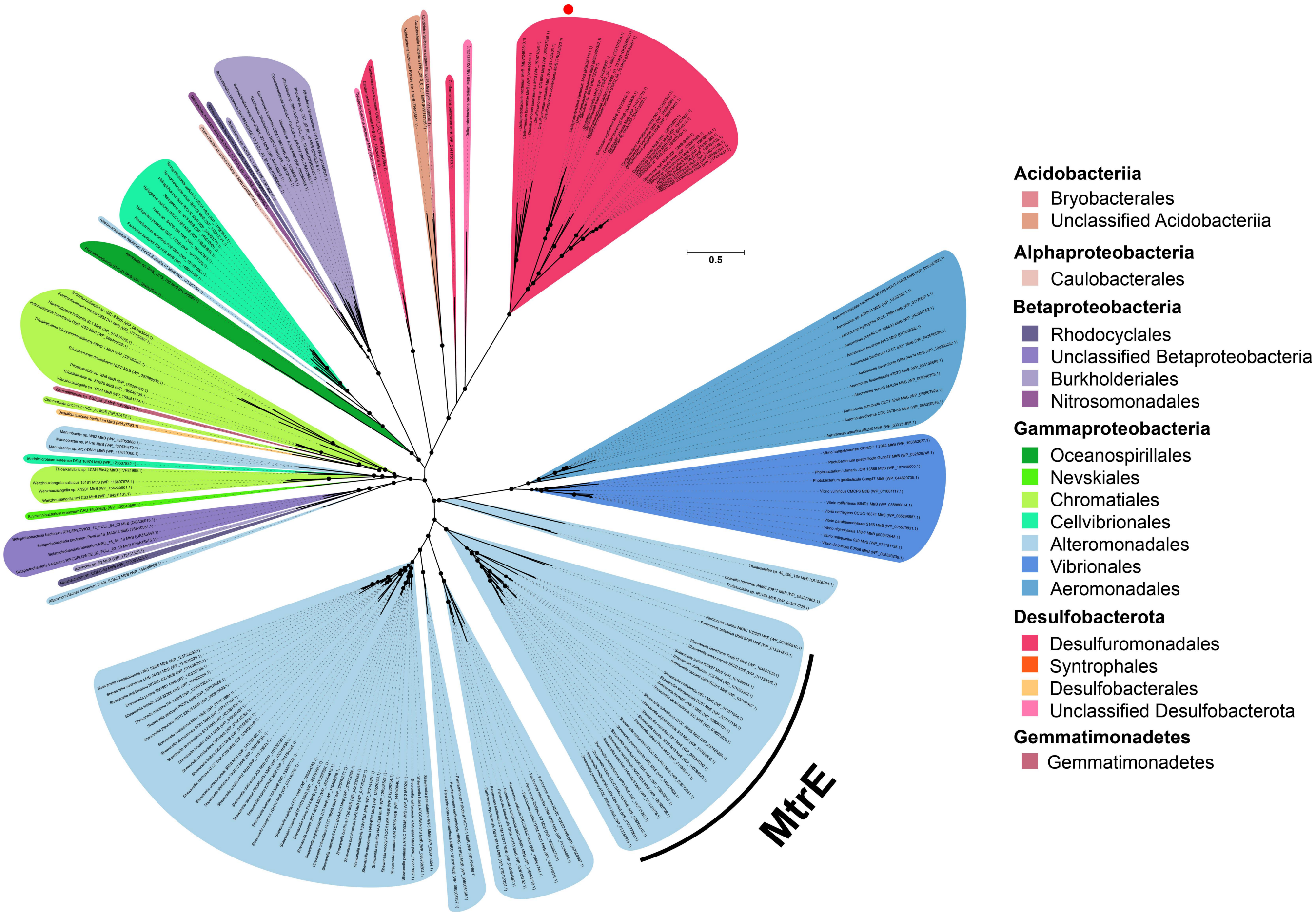

Supplementary Fig. 16 Phylogenetic relationship among known MtrB from diverse bacteria and the novel *Desulfobacterota* MtrB detected in this study. Maximum-likelihood phylogenetic tree constructed from a concatenated alignment of the known diversity of MtrB β-barrel and the novel *Desulfobacterota* MtrB detected in this study. Color codes were assigned by taxonomic order. Bootstrap support values ≥90% from 1000 resamples are indicated with filled black dots. The scale bar represents average changes per amino acid position. MtrE indicates homologs of MtrB. MtrB of *D. acetexigens* is indicated with a red dot.
